# Anti-inflammatory, Anti-fibrotic and Pro-cardiomyogenic Effects of Genetically Engineered Extracellular Vesicles Enriched in miR-1 and miR-199a on Human Cardiac Fibroblasts

**DOI:** 10.1007/s12015-023-10621-2

**Published:** 2023-09-13

**Authors:** Katarzyna Kmiotek-Wasylewska, Sylwia Bobis-Wozowicz, Elżbieta Karnas, Monika Orpel, Olga Woźnicka, Zbigniew Madeja, Buddhadeb Dawn, Ewa K. Zuba-Surma

**Affiliations:** 1 Department of Cell Biology, Faculty of Biochemistry, Biophysics and Biotechnology, Jagiellonian University, Gronostajowa 7, 30-387 Krakow, Poland; 2 Department of Cell Biology and Imaging, Institute of Zoology and Biomedical Research, Jagiellonian University, Gronostajowa 7, 30-387 Kraków, Poland; 3 Department of Internal Medicine, Kirk Kerkorian School of Medicine at the University of Nevada, Las Vegas, 1701 W Charleston Blvd., Las Vegas, NV 89102 USA

**Keywords:** Extracellular vesicles, Induced pluripotent stem cells, miR-1, miR-199a, Cardiac fibroblasts, Heart repair

## Abstract

**Rationale:**

Emerging evidence indicates that stem cell (SC)- derived extracellular vesicles (EVs) carrying bioactive miRNAs are able to repair damaged or infarcted myocardium and ameliorate adverse remodeling. Fibroblasts represent a major cell population responsible for scar formation in the damaged heart. However, the effects of EVs on cardiac fibroblast (CFs) biology and function has not been investigated.

**Objective:**

To analyze the biological impact of stem cell-derived EVs (SC-EVs) enriched in miR-1 and miR-199a on CFs and to elucidate the underlying molecular mechanisms.

**Methods and Results:**

Genetically engineered human induced pluripotent stem cells (hiPS) and umbilical cord-derived mesenchymal stem cells (UC-MSCs) expressing miR-1 or miR-199a were used to produce miR-EVs. Cells and EVs were thoughtfully analyzed for miRNA expression using RT-qPCR method. Both hiPS-miRs-EVs and UC-MSC-miRs-EVs effectively transferred miRNAs to recipient CFs, however, hiPS-miRs-EVs triggered cardiomyogenic gene expression in CFs more efficiently than UC-MSC-miRs-EVs. Importantly, hiPS-miR-1-EVs exhibited cytoprotective effects on CFs by reducing apoptosis, decreasing levels of pro-inflammatory cytokines (CCL2, IL-1β, IL-8) and downregulating the expression of a pro-fibrotic gene – α-smooth muscle actin (α-SMA). Notably, we identified a novel role of miR-199a-3p delivered by hiPS-EVs to CFs, in triggering the expression of cardiomyogenic genes (*NKX2.5, TNTC, MEF2C*) and ion channels involved in cardiomyocyte contractility (*HCN2, SCN5A, KCNJ2, KCND3*). By targeting SERPINE2, miR-199a-3p may reduce pro-fibrotic properties of CFs, whereas miR-199a-5p targeted *BCAM* and *TSPAN6*, which may be implicated in downregulation of inflammation.

**Conclusions:**

hiPS-EVs carrying miR-1 and miR-199a attenuate apoptosis and pro-fibrotic and pro-inflammatory activities of CFs, and increase cardiomyogenic gene expression. These finding serve as rationale for targeting fibroblasts with novel EV-based miRNA therapies to improve heart repair after myocardial injury.

**Graphical Abstract:**

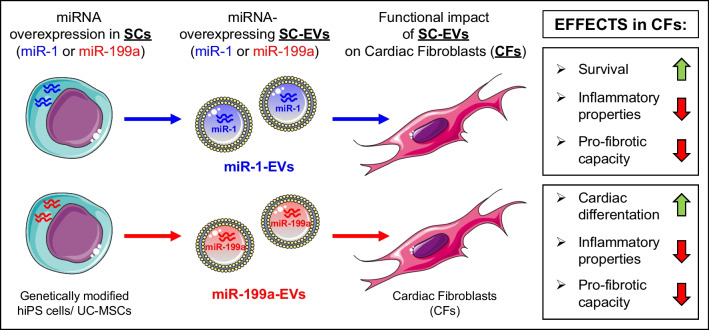

**Supplementary Information:**

The online version contains supplementary material available at 10.1007/s12015-023-10621-2.

## Introduction

Cardiovascular diseases remain the major cause of mortality and morbidity worldwide, raising serious socio-economic challenges [1]. Cardiomyocytes (CMs) loss and their replacement by fibroblasts forming a scar are the main consequences of heart injury or myocardial infarction (MI). Recent advances in cardiac cell-based therapies rely on injection of either CMs or various populations of stem cells, with the goal to reconstitute functional cardiac tissue [2]. However, the level of stem cells engraftment remains usually very low due to also unfavourable post-ischemic environment [3]. The inflammatory response to ischemia–reperfusion is a critical factor determining the size of the infarct zone and the extent of adverse heart tissue remodelling [4]. The key players in triggering inflammation in the post-ischemic heart are dying cardiomyocytes and endothelial cells, which release necrotic factors, and cardiac fibroblasts (CFs). Importantly, CFs constitute almost 20% of heart cells and they actively participate in the process of tissue remodelling after CMs damage [5]. By secretion of pro-inflammatory cytokines they attract immune cells to the site of injury and upon fibroblast-to-myofibroblast transition (FMT), they are responsible for heart tissue fibrosis [6, 7]. Thus, modulating inflammatory and pro-fibrotic properties of CFs seems to be an attractive target in the future cardiovascular therapies.

Apart from direct differentiation of stem cells towards CMs, recent studies have pointed towards important role of stem cells’ paracrine activity during heart regeneration [8]. In this regard, the role of extracellular vesicles (EVs) secreted by all eukaryotic cells, is particularly important [9]. EVs are composed of a cellular membrane enclosing bioactive cytosolic cargo, including proteins, lipids and nucleic acids (mainly RNA, such as mRNA, microRNA; miRNA and long non-coding RNA; lncRNA). Role of stem cell-derived EVs in cardiac tissue repair has been investigated in several pre-clinical animal models. In particular, embryonic stem cells (ESCs), induced pluripotent stem cells (iPS), mesenchymal stem cells (MSCs), cardiac progenitor cells (CPCs) and CD34-positive hematopoietic stem cells (HSCs) were used as a source of reparative EVs [10–12]. In our previous work, we demonstrated that injection of murine iPS cell- derived EVs ameliorated adverse remodeling after acute MI in mice, reduced CM apoptosis and enhanced angiogenesis [13]. Collectively, these results showed significant beneficial effects of stem cell-EV-based therapy in heart regeneration.

It is now believed that one of the major determinants of EVs’ effects on target cells represents small RNA molecules. Among them, micro-RNAs (miRNAs) have gained particular interest, as epigenetic modifiers of cellular function. miRNAs important for CM homeostasis have been described, including miR-1 and miR-133 involved in CM differentiation; miR-199a, miR-590, miR-17–92 cluster driving CM proliferation; and miR-21, miR-24 protecting from apoptosis [14–16]. However, potential roles of these miRNAs on CFs, which represent a major cellular population in the myocardium, has not been examined. Interestingly, serum level of miR-1 is increased after MI [17] and miR-1 overexpression prevents heart tissue from fibrosis [18] and hypertrophy [19, 20]. Further, miR-199a has been implicated in specification of mesodermal lineages [21, 22] including maturation of embryonic stem cell-derived CMs [23], and its delivery to infarcted hearts improved cardiac function [24, 25]. Thus, we hypothesized that miR-1 and miR199a are involved in the regulation of CF fate decisions and biological activities.

To verify our hypotheses, we genetically engineered human iPS cells and umbilical cord-derived MSC (UC-MSC) to stably express miR-1 and miR-199a. EVs released by parental cells were collected and used to study miR-1 and miR-199a effects on CFs. We first validated efficacy of miRNA transfer by hiPS and UC-MSC-derived EVs to CFs and their ability to change fate decision toward cardiomyocytes. We found stronger induction of cardiomyogenic genes after hiPS-miRs-EVs treatment, in comparison with the activity of UC-MSC-miR-EVs. Next, we experimentally tested miRNAs-EV role in CF biological functions, and observed decreased proliferation, increased metabolic activity after treatment with miR-1/miR-199a-EVs; decreased apoptosis, particularly after miR-1-EVs; and enhanced spontaneous cardiomyogenic differentiation post miR-199a-EVs treatment. Finally, we sought to identify molecular mechanisms underlying miRNA-EVs-mediated effects on CFs. For the first time, we demonstrated anti-apoptotic, anti-inflammatory and anti-fibrotic role of miR-1-EVs on CFs. Moreover, we identified a role of miR-199a-3p in the induction of cardiomyogenesis in CFs, and possible involvement in attenuation of inflammation and reduction of fibrosis. These findings may be utilized to develop EV-based novel therapies to ameliorate adverse remodeling after myocardial injuries and improve heart function in patients with heart diseases.

## Methods

### Cell Culture

UC-MSCs were maintained according to the approval of local ethical committee. Umbilical cord was provided by the Polish Stem Cell Bank (Warsaw, Poland) accordingly to required legal approvals and procedures hold by PBKM. UC-MSCs were isolated using an explant method described previously [26]. Cells were cultured in DMEM/F12 (Gibco/Thermo Fisher Scientific, Waltham, MA, USA) supplemented with 10% fetal bovine serum (FBS; Sigma-Aldrich/Merck, St. Louis, MO, USA) and penicillin (100 U/ml), streptomycin (100 μg/ml) solution (P/S; Gibco).

CFs were purchased from Lonza (Normal Human Cardiac Fibroblasts – Ventricular; NHCF-V; Lonza, Basel, Switzerland) and were cultured in DMEM/F12 (Sigma-Aldrich) containing 15% FBS (Sigma-Aldrich) and P/S (Gibco).

hiPS cells generated in our laboratory [27] were cultured in feeder- serum- and xeno-free conditions in Essential 8 medium (Gibco) supplemented with P/S (Gibco) on rhVitronectin (50 μg/ml; Gibco) coated plates. Every four days cells were re-seeded on new plates using 0.5 mM EDTA (Invitrogen/Thermo Fisher Scientific) and supplemented with 10 µM/ml Rho-associated protein kinase inhibitor (Y-27632, Merck) for the first day.

### Lentivirus Production and Generation of Stable Cell Lines Expressing miRNAs

Lentiviral expression vectors for human pre-miR-1 and pre-miR-199a (pHMIR-1 and pHMIR-199a) were purchased from Systems Biosciences (Palo Alto, CA, USA). Control vector expressing green fluorescent protein (copGFP) was created by deleting DNA fragment containing CMV7 promoter with miRNA sequence from pHMIR-1 plasmid by Spe I and Not I digestion (both from New England Biolabs), blunting DNA ends with the Quick blunting Kit (New England Biolabs, Ipswich, MA, USA) and re-ligation with the T4 DNA Ligase (Thermo Fisher Scientific).

Lentiviral vector particles were produced in HEK293T/17 packaging cell line (ATCC-CRL-11260; LGC Standards, Teddington, UK), by co-transfection of miRNA/copGFP-expression plasmid with psPAX2 and pMD2G packaging plasmids (#12260 and #12259, respectively; Addgene, Cambridge, MA, USA), using Lipofectamine2000 (Invitrogen/Thermo Fisher Scientific) as a transfection agent. Cell supernatants were filtered through 0.2 µm pores PVDF filters (Millipore/Merck) and used for infection of hiPS cells and UC-MSCs with MOI of 5 in Essential 8 medium or DMEM/F12 supplemented with 5% FBS, respectively, and with addition of 10 µg/1 mL of polybrene (Milipore). After culture expansion for at least 6 days, cells expressing copGFP were sorted using the cell sorter FACS Aria III (BD Biosciences, San Jose, CA, USA).

### EV Isolation

At 70–90% confluency, conditioned medium was collected form cultures of control wild type (hiPS-WT/UC-MSC-WT), control copGFP- expressing (hiPS-copGFP/UC-MSC-copGFP) and miRs- overexpressing (hiPS-miRs/UC-MSC-miRs) cells for extracellular vesicles isolation. UC-MSC-miRs were washed twice with PBS to remove FBS and for EV collection cells were kept in DMEM/F12 supplemented with 0.5% bovine serum albumin (BSA; Sigma-Aldrich) and P/S for 48 h. EVs from iPS cell lines were directly harvested from the serum- free medium used for cell culture. EVs were isolated according to sequential centrifugation protocol, as previously described [26, 27]. The obtained EV pellets were re-suspended in 150 μL of PBS (Lonza). Protein concentration was determined with the Bradford assay.

### Nanoparticle Tracking Analysis (NTA)

Concentration and size distribution of EVs was measured with the NanoSight NS300 nanoparticle analyzer (Malvern, Worchestershire, UK). All data were collected with the camera level of 13 and the detection threshold of 3. Samples were diluted in PBS 1:1000 for optimal particle count (1 × 10^8^ to 1 × 10^10^). Using the script control function, three 60-s videos were recorded for each sample.

### Transmission Electron Microscopy (TEM)

20 µM of EV suspension in PBS was added to nickel grids (Agar Scientific, Stansted, UK) for 30 min. EVs were fixed for 5 min with 2,5% glutaraldehyde solution. The grid was blotted with filter paper and stained with 2% uranyl acetate for 2 times for 30 min. Next, grids were washed in distilled water 3 times for 1 min. Grids was dried and EVs were visualizes by JEOL JEM2100 HT CRYO LaB6 transmission electron microscope (JEOL, Peabody, MA, USA).

### EV Incubation with CFs

CFs were seeded on 24-well plates in DMEM/F12 medium supplemented with 15% FBS and P/S. After 24 h, cells were treated with EVs (20 ng/1000 cells) for 3 h. After indicated time points, cells were washed twice with PBS and collected for analysis.

### Cardiac Differentiation

CFs were subjected to two distinct cardiac differentiation protocols. In both, 5 × 10^4^ cells were seeded on 12-well plates in DMEM/F12 with 15% FBS (Sigma-Aldrich) and P/S. After 1 day cells were treated with EVs (20 ng/1000 cells) for 24 h. In the first protocol cells were treated with differentiation medium, composed of DMEM/F12 supplemented with 2% FBS and 10 ng/mL bFGF, 10 ng/mL VEGF, and 10 ng/mL TGFβ1 (all growth factors from PeproTech, London, UK). In the second protocol, EV-treated cells were cultured in DMEM/F12 supplemented with 2% FBS. Control cells were placed in differentiation medium (described above). The medium was changed every day in both protocols. After 7 days, cells were collected for analysis.

### Proliferation

The rate of CF proliferation was examined using the Cell Counting Kit-8 (Sigma) according to vendor’s instruction. CFs were seeded in DMEM/F12 medium supplemented with 15% FBS and P/S. After 24 h, cells were treated with EVs (20 ng/1000 cells) for 24 h. Proliferation was evaluated at 24, 48 and 96 h after EV treatment in cells cultured in standard conditions (21% O_2_) or in hypoxia (1% O_2_). Absorbance was measured using Multiskan FC Microplate Photometer (Thermo Fisher).

### Metabolic Activity Analysis

ATP production was measured with the ATPLite Kit (PerkinElmer Waltham, MA, USA) according to manufacturer’s protocol. CFs were seeded in DMEM/F12 medium supplemented with 15% FBS and P/S and after 24 h, cells were treated with EVs for the next 24 h. Metabolic activity was evaluated at 24, 48 and 96 h after EV treatment in CFs cultured in normoxia (21% O_2_) or hypoxia (1% O_2_). Luminescence was measured using the Infinite M200 Microplate Reader (Tecan, San Jose, CA, USA).

### Apoptosis Analysis

Apoptosis was examined using the CellEvent Caspase-3/7 Green Detection Reagent (Thermo Fisher) in two experiments. First, to verify whether EV pretreatment induces cytoprotective benefits, CFs were seeded on 6-well plates, and after 24 h treatment with EVs (20 ng/1000 cells), 1 μM of staurosporine (Santa Cruz Biotechnology, Dallas, TX, USA) was added for 6 h (marked as EVs- > S). Second, to examine whether EV treatment can salvage cells from apoptosis after an injury, CFs were first treated with 1 μM of staurosporine for 6 h followed by EVs addition (20 ng/1000 cells) for 24 h (marked as S- > EVs). Fluorescence was measured on LSR Fortessa flow cytometer (BD Biosciences).

### Treatment of CFs with miRNA Inhibitors

CFs were first treated with EVs (20 ng/1000 cells) for 24 h, and then electroporated using the Neon Transfection System (Thermo Fisher Scientific) with the following electric pulse parameters: 1400 V, 20 ms, 3 pulses, to introduce miRNA inhibitors (miR-1-3p, miR-199a-3p and miR-199a-5p from Exiqon/Qiagen or control inhibitor from Qiagen, Germantown, MD, USA) at 50 nM final concentration per 50,000 cells. After 48 h cells were collected for molecular analysis. One experimental sample was composed of cells from 3 nucleofections. To assess cardiac differentiation of CFs treated with miR-199a-EVs, cells after nucleofection were kept in DMEM/F12 supplemented with 2% FBS for 7 days with daily medium change. For apoptosis assessment in CFs treated with miR-1-EVs, 24 h post-nucleofection 1uM of staurosporine was added for 6 h and then cells were collected for analysis.

### Quantitative Real-time PCR Analysis

Total RNA was isolated from cellular or EV samples using the GeneMATRIX Universal RNA/miRNA Purification Kit (Eurx, Gdansk, Poland) and used for cDNA synthesis with the NG dART RT Kit (Eurx) for mRNA analysis or with the Universal cDNA synthesis Kit II (Exiqon) for miRNA analysis, according to the manufacturers’ instructions. Transcript levels were measured using the real-time PCR method with the SYBR Green Master Mix (Applied Biosystems/Thermo Fisher Scientific) and specific primer sets listed in the Supplementary Table 1 or purchased from the Real Time Primers (Elkins Park, PA, USA) for miRNA target screen. For miRNA expression Power SYBR Green Master Mix (Applied Biosystems/Thermo Fisher Scientific) was used with specific LNA-primers (Exiqon). Quantification of mRNA and miRNA content was performed on the QuantStudio 6 Fast Real-Time PCR System (Applied Biosystems/Thermo Fisher Scientific) using the ∆∆Ct method with β-2-microglobulin as endogenous control for mRNA and U6 snRNA for miRNA analysis. The data were normalized when compared to control (WT) cellular or EV samples.

### Western Blot

Genetically modified and unmodified hiPS cells, UC-MSCs and EVs derived from these cells were lysed in RIPA buffer (Thermo Fisher Scientific) containing proteinase inhibitors. EVs were lysed in 3:1 ratio. 30 µg of protein extracts were separated by Mini-PROTEAN TGXPrecast Gels (BioRad, Hercules, CA, USA) and transferred to PVDF membranes using Trans-Blot Turbo RTA Mini PVDF Transfer Kit (BioRad). Proteins were detected with the mouse monoclonal exosome anti-CD9 (10626D, Invitrogen), goat polyclonal anti-Syntenin/SDCBP (PA5-18595, Invitrogen), goat polyclonal anti-Calnexin (PA5-19169, Invitrogen), mouse monoclonal anti-alpha smooth muscle actin (A2547, Sigma), mouse monoclonal anti-Desmin (D1033), and rabbit polyclonal anti-Cx43 (C6219, Sigma) antibodies. Equal loading was evaluated by staining the samples with the mouse monoclonal IgG β-actin (sc-81178, Santa Cruz Biotechnology) or mouse monoclonal anti-beta tubulin (T4026, Sigma) antibodies. The proteins were detected with horse radish peroxidase (HRP)-conjugated rabbit anti-goat IgG (H + L) (R21459, Invitrogen), goat anti-mouse IgG, IgM (H + L) (31444, Invitrogen) or goat anti-rabbit IgG (H + L) (A27036, Invitrogen) secondary antibodies. The membranes were developed with Luminata Crescendo Western HRP Substrate (Merck) and imaged by Gel Doc XR + Gel Documentation System (Bio-Rad).

### Proteome Arrays

The Human Phospho Kinase Protein Array kit (ARY003B) and the Human Apoptosis Array Kit (ARY009) (both from R&D Systems, Minneapolis, MN, USA) were used to evaluate the relative protein levels in CF treated with EV-miRs and with respective inhibitors. The procedures were performed according to the vendor’s recommendation. Chemiluminescent signals were detected by the ChemiDoc XRS + System (BioRad). Pixel density was measured using the Quantity One software (BioRad) by an independent researcher, who did not perform the experiments and was blinded to the samples names.

### Statistical Analysis

At least two experiments were performed in duplicate for each study. The data are presented as means ± standard deviations (SD). Statistical analyses were done with unpaired Student’s t-test or one-way ANOVA and Tukey’s multiple comparisons test using GraphPad Prism7 (GraphPad Software, La Jolla, CA, USA). *p* value of < 0.05 was considered statistically significant (* *p* < 0.05; ** *p* < 0.01; *** *p* < 0.001).

## Results

### Genetically Modified Stem Cell Lines Express High Levels of Transgenic miRNAs

**Fig. 1:**
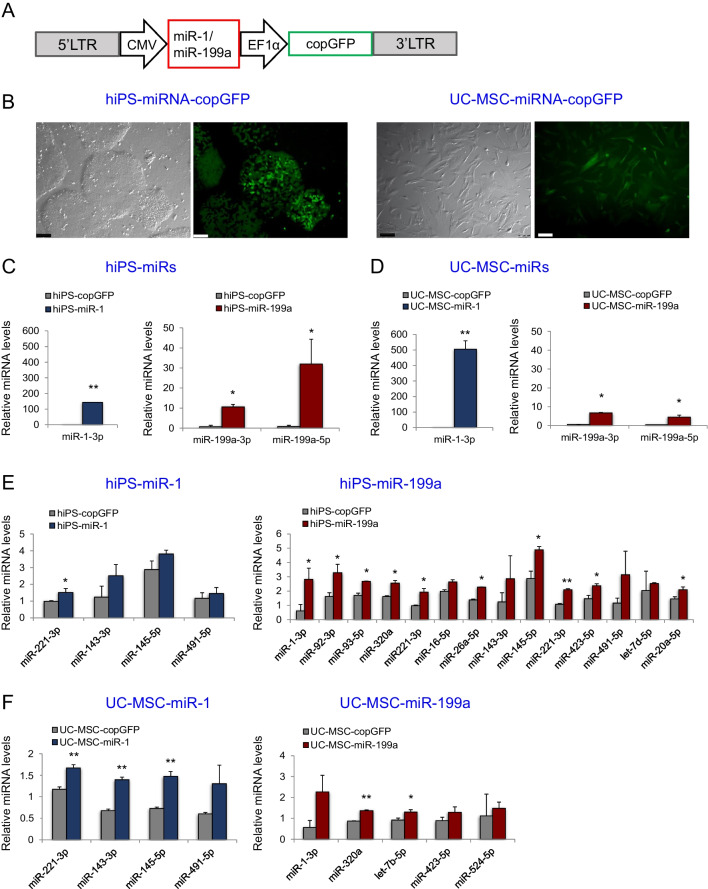
Generation of transgenic stem cell lines overexpressing miR-1 and miR-199a. (**A**) Scheme of cassette harbouring miR-1 or miR-199a coding sequence in lentiviral vector used for transduction. LTR, long terminal repeat; CMV, cytomegalovirus promoter; EF1α, elongation factor 1 alpha gene promoter; copGFP, variant of a green fluorescent protein derived from copepepod *Pontellina plumata.* (**B**) Representative microscopic images of hiPS and UC-MSC genetically modified cells overexpressing miR-1 or miR-199a (co-expressing copGFP in green). Scale bar = 100 μm. Relative miRNA expression levels of miR-1 or miR-199a in genetically engineered hiPS cells (**C**) and UC-MSCs (**D**). Expression levels of miRNAs co-induced after miR-1 or miR-199a overexpression in hiPS cells (**E**) and UC-MSCs (**F**). The data on panels C-F were normalized compared with the respective control (WT) cellular samples. Student’s t-test; comparison with copGFP-control cell lines; *p < 0.05, **p < 0.01

Since the major goal of this study was to use EVs derived from stem cells as carriers of selected miRNAs to improve CF function, we first generated stable stem cell lines expressing miR-1 and miR-199a. We used hiPS cell line generated in our laboratory [27] and UC-MSCs, since unmodified EVs from both stem cell populations showed efficacy in ameliorating myocardial infarction symptoms [28]. To test the hypothesis that pro-cardiomyogenic miRNAs loaded into EVs may further boost protective effects exerted on heart cells, hiPS cells and UC-MSCs were transduced with lentiviral vectors expressing miR-1 or miR-199a (Fig. 1A). The vector additionally contained a marker protein, copGFP, for easy selection and monitoring of transgene expression in cells (Fig. 1B). Next, we measured miRNAs levels in genetically modified stem cell populations and control cells: unmodified (wild type; WT) and copGFP cells. We detected high expression levels of both miR-1 and miR-199a in respective hiPS cells and UC-MSCs (Fig. 1C and D, respectively). Moreover, to verify the impact of upregulated miRNAs on the global miRNome in the engineered cells, we screened the expression levels of the 44 most abundant miRNAs detected in hiPSCs and UC-MSCs. Our analysis revealed that expression of miR-1 and miR-199a led to upregulation of other miRNAs both in hiPS cells and UC-MSCs (Fig. 1E and F, respectively). Notably, hiPS-miR-199a transduced cells displayed the highest number of co-induced miRNAs (Fig. 1E).

### EVs Serve as Carriers of Transduced miRNAs

**Fig. 2:**
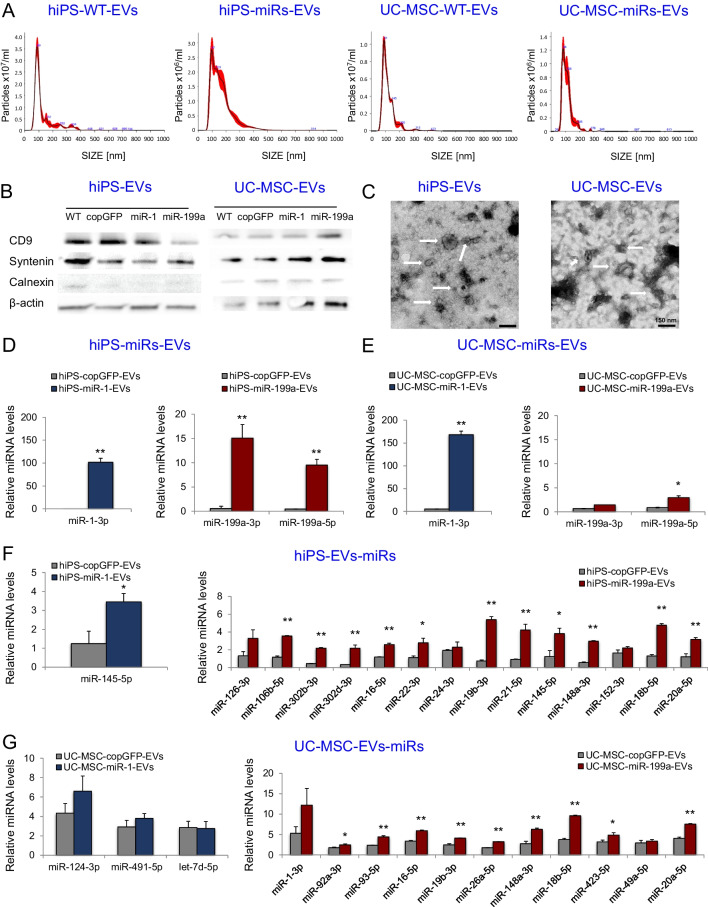
Characterization of EVs from hiPS cells and UC-MSCs stably expressing miR-1 or miR-199a. (**A**) Particle size distribution in EV samples derived from control and genetically modified stem cells, measured by nanoparticle tracking analysis (NTA)*.* Representative histograms are shown. (**B**) Western blot analysis of selected proteins in hiPS-miRs-EVs and UC-MSC-miRs-EVs. (**C**) Representative images of EVs obtained by transmission electron microscopy. Scale bars = 150 nm. (**D**) Relative levels of miR-1 and miR-199a in hiPS-miR-1-EVs and hiPS-miR-199a-EVs, respectively. (**E**) Relative levels of miR-1 and miR-199a in UC-MSC-miR-1-EVs and UC-MSC-miR-199a-EVs, respectively. (**F**) Other miRNAs found at elevated levels in hiPS-miR-1-EVs or hiPS-miR-199a-EVs, when compared with hiPS-WT control. (**G**) Other miRNAs found at elevated levels in UC-MSC-miR-1-EVs or UC-MSC-miR-199a-EVs, when compared with UC-MSC-WT control. The data on panels D-G were normalized compared with the respective control (WT) EV samples. Student’s t-test; comparison with copGFP-control EVs; *p < 0.05, **p < 0.01

To verify whether transduced miRNAs can be exported from parental cells to EVs, we isolated EVs from conditioned media collected from genetically modified and control stem cell lines, using the sequential ultracentrifugation method. Particle distribution analysis with NTA revealed that the mean EV size was in a range of 110 – 120 nm in diameter (Fig. 2A and Supplementary Table 2). EVs were then examined for the presence of typical proteins, according to the criteria launched by the International Society of Extracellular Vesicles (ISEV) [29]. Western blot analysis showed that EVs from genetically engineered and control stem cell lines expressed a membrane-bound tetraspanin CD9, intraluminal syntenin, low level of endoplasmic reticulum-associated calnexin, and control protein, β-actin (Fig. 2B). Expression of these proteins was also analyzed in parental cells, which is shown in the Supplementary Fig. 1. EVs were then visualized by TEM (Fig. 2C and Supplementary Fig. 2). Finally, we analyzed miRNAs levels in EVs from genetically modified and unmodified stem cell lines and we observed substantial enrichment in EVs of the respective overexpressed miRNAs (Fig. 2D, E). However, variable level of miRNAs content between hiPS cell- and UC-MSC- derived EVs suggests selective packaging of miRNAs into the EVs, depending on cell type. Similarly, as in parental cells, overexpression of certain miRNA led to global changes in miRNome profile in EVs (Fig. 2F, G), in particular in case of miR-199a-enriched EVs. To investigate the potential role of upregulated miRNAs in hiPS-miR-199a-EVs and UC-MSC-miR-199a-EVs, we performed pathway analysis using the DIANA-miRPath v3.0 online software [30]. Importantly, the screen revealed regulatory role of miR199a-EVs derived from both stem cell populations in TGFβ (transforming growth factor β) signaling, FoxO (Forkhead box O) signaling, Hippo pathway, extracellular matrix (ECM)-receptor interactions, regulation of pluripotency (Supplementary Fig. 3 and Fig. 4).

### hiPS-EVs Outperform UC-MSC-EVs in Inducing Cardiomyogenesis

**Fig. 3:**
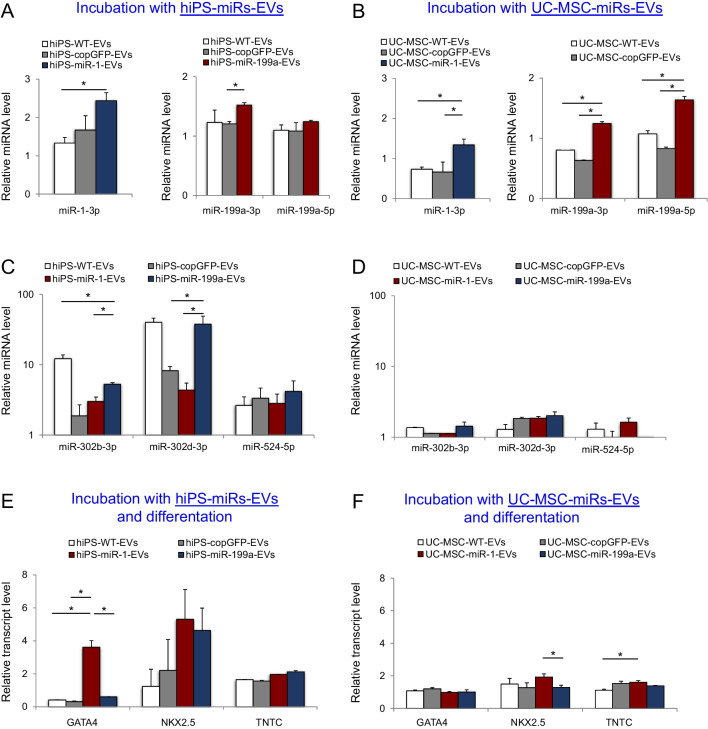
Treatment of cardiac fibroblasts with EVs derived from genetically modified stem cells*.* Expression levels of miR-1 and miR-199a in CFs after incubation with hiPS-miR-1-EVs and miR-199a-EVs, respectively (**A**); or UC-MSC-miR-1-EVs and miR-199a-EVs, respectively (**B**). Expression levels of pluripotency-associated miRNAs in CFs after incubation with EVs derived from genetically modified hiPS cells (**C**) and UC-MSCs (**D**). Expression levels of cardiomyogenic genes in CFs treated with hiPS-miRs-EVs (**E**) or UC-MSC-miRs-EVs (**F**) and subjected to induction of cardiac differentiation for 7 days. The data were normalized compared with control samples from CFs without EV treatment. ANOVA multiple comparisons and Tukey test; *p < 0.05, **p < 0.01

We tested the efficacy of EVs derived from the two stem cell populations in miRNA transfer to CFs. After 3 h of incubation with miRNA-EVs, CFs were analyzed for the expression of selected miRNAs. Our results confirmed that both hiPS-EVs and UC-MSC-EVs serve as good vehicles for miRNA delivery to CFs, leading to 1.5 – twofold increase in miR-1 and miR-199a levels (Fig. 3A, B). Notably, hiPS-EVs co-transferred pluripotency-associated miRNAs (miR-302b, miR-302d [31], miR-524-5p [32]; Fig. 3C), which was not observed with UC-MSC-EVs treatment (Fig. 3D). Based on our previous findings indicating enhanced cardiac differentiation of human mesenchymal stromal cells after hiPS-EVs treatment [27], we tested efficiency of cardiomyogenesis induction in CFs after hiPS-miRs-EVs and UC-MSC-miRs-EVs. Our results show elevated transcript levels of genes involved in cardiac lineage specification (*GATA4, NKX2.5, TNTC*) particularly after hiPS-miRs-EVs (Fig. 3E). Although incubation with UC-MSC-miRs-EVs followed by cardiac differentiation of CFs led to increased expression of *NKX2.5* and *TNTC*, the transcript levels were substantially lower than after hiPS-miRs-EVs (Fig. 3F). Moreover, during culture and expansion of stem cell populations expressing miRNAs, we observed remarkably higher proliferative ratio for miR-transduced hiPS cells, in comparison to miR-transduced UC-MSCs. This observation was supported by the detection of higher mRNA levels for anti-apoptotic gene *BCL2*, regulators of proliferation (*c-MYC*) and cell cycle progression (*CDK2, CCNE1*) in miR-transduced hiPS cells (Supplementary Fig. 5A). On the contrary, miR-transduced UC-MSCs exhibited higher level of pro-apoptotic gene *BAX* and cell cycle inhibitors (*p21, CCND1*; Supplementary Fig. 5B). Considering that large quantities of cell supernatant are required for EV isolation, we chose miR-transduced hiPS cell lines as superior EV-producer cells for further studies.

### hiPS-miRNA-EVs Decrease Proliferation and Increase ATP Levels in CFs

**Fig. 4:**
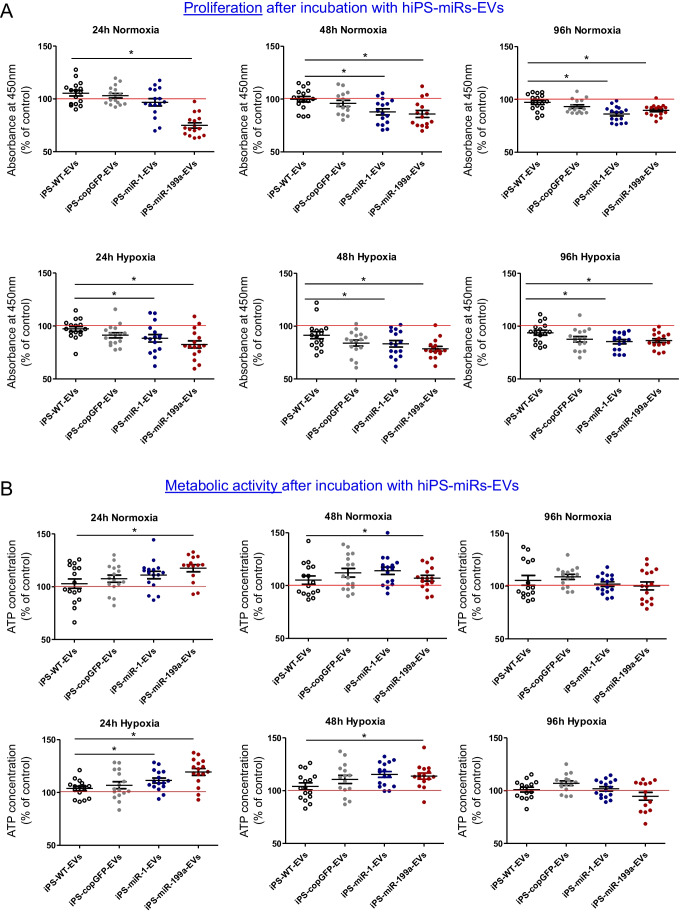
Proliferation and metabolic activity of cardiac fibroblasts after EVs treatment. Analysis of proliferation capacity (**A**) and metabolic activity (**B**) of CFs following EV treatment for 24 h. The analyses were performed at 24, 48 and 96 h after incubation with EVs on CFs cultured in normoxic (upper panels) or hypoxic (1% O_2_; lower panels) conditions. The data were normalized compared with control CFs without EV treatment; red line. Student’s t-test; *p < 0.05

To analyze biological impact of hiPS-miRNAs-EVs on target cells, CFs were subjected to proliferation and metabolic activity tests upon miRs-EVs treatment. The assays were performed in normoxic (21% O_2_) or hypoxic condition (1% O_2_), the latter of which mimiced deleterious environment within the ischemic tissue. We observed decreased proliferation of CFs after hiPS-miR-1-EVs and hiPS-miR-199a-EVs, in both, ambient and reduced oxygen conditions (Fig. 4A). On the contrary, ATP production, which is a surrogate of metabolic activity, was elevated in CFs after hiPS-miR-1-EVs and hiPS-miR-199a-EVs treatment in normoxia or hypoxia, at 24 and 48 h post-treatment (Fig. 4B). This effect declined at 96 h post-treatment, indicating transient effect mediated by hiPS-miRs-EVs on CFs. Interestingly, we also observed enhanced migratory activity of CFs upon stimulation with hiPS-miRs-EVs in terms of speed and the distance (Supplementary Fig. 6). However, the observed effects were not specific to miR-1 or miR-199a since control hiPS-copGFP-EVs stimulated migration of CFs at the same level as hiPS-miR-1-EVs and hiPS-miR-199a-EVs (Supplementary Fig. 6).

### Antiapoptotic and Cardiomyogenic Effects hiPS-miR-1-EV and hiPS-miR-199a-EV on CFs

**Fig. 5:**
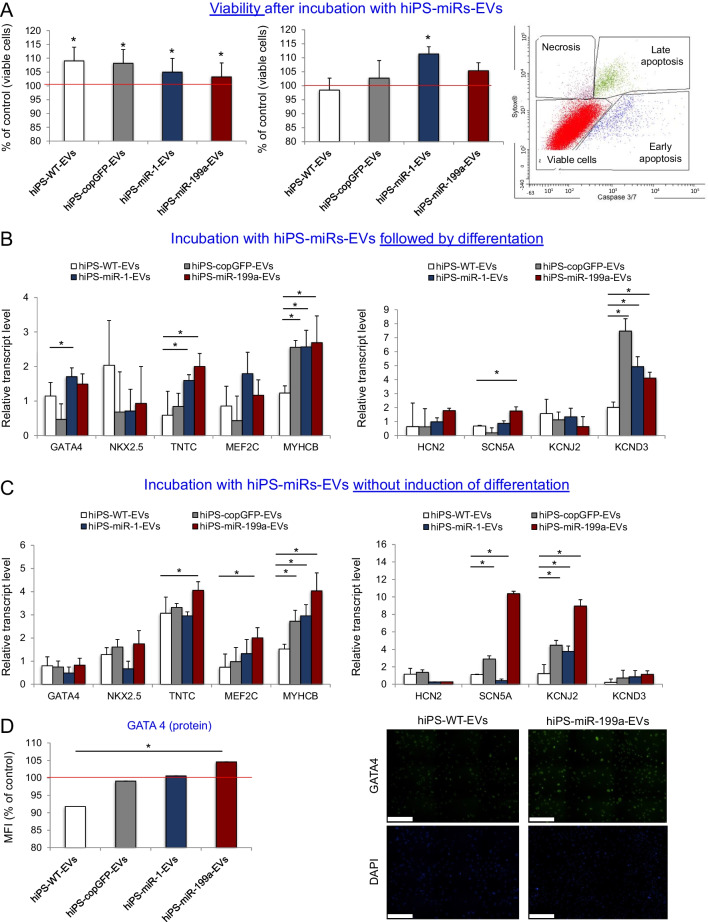
Impact of hiPS-EVs on survival and activation of cardiomyocytic gene expression in CFs *in vitro*. (**A**) Flow cytometric analysis of CF viability and survival after incubation with iPS-EVs following (left graph) or followed by (right graph) treatment with cytotoxic agent (staurosporine) inducing cell apoptosis. The data were normalized compared with the control CFs without EV treatment; red line. Representative dot-plot from flow cytometric analysis is shown to demonstrate the gating strategy. Real time qPCR analysis of expression of selected genes indicative of cardiomyogenic differentiation and cardiac ion channels in CFs after treatment with EVs followed by cardiac differentiation (**B**) or without induction of differentiation (**C**) at 7 day of culture. The data were normalized compared with control CFs without EV treatment. (**D**) Quantitative (left panel) and qualitative (right panel) analysis of GATA4 protein expression in CFs after iPS-EV treatment. Representative fluorescence tile scan images are shown for CFs treated with hiPS-WT-EVs and hiPS-miR-199a-EVs. The composite of nine (3 × 3) title scans for every image are shown. Scale bar = 100μm. Student’s t-test; *p < 0.05

To further examine the impact of miR-1-EVs and miR-199a-EVs on CFs, target cells were subjected to apoptosis and cardiomyogenesis stimulating assays. Cytoprotective and anti-apoptotic properties of EVs were evaluated in staurosporine-induced cell death experiments. First, to study cytoprotection, CFs were co-incubated with hiPS-miRs-EVs for 24 h, followed by the drug treatment for 6 h and enumeration of live and apoptotic events by flow cytometry (Fig. 5A). Impressively, pretreatment with all types of hiPS-miRs-EVs and control EVs exerted strong cytoprotective effect on CFs, as evidenced by increased percentage of live cells (Fig. 5A, left). Second, to verify anti-apoptotic ability of hiPS-miRs-EVs, CFs were first subjected to staurosporine for 6 h first, after which EVs were added to the culture for the next 24 h. In this scenario, significant improvement of cell survival was detected only for hiPS-miR-1-EVs (Fig. 5A, middle).

Knowing that the transfer of bioactive cargo of hiPS-EVs to recipient cells may trigger lineage specification, we analyzed expression of cardiomyogenesis-related genes in CFs upon treatment with hiPS-miRs-EVs. Following a differentiation protocol based on the use of cardiomyogenic medium, we observed a significant upregulation of *TNTC* transcript level after treatment with hiPS-miR-1-EVs and hiPS-miR-199a-EVs (Fig. 5C, left). We also noted strong increase in *MYHCB* transcript level, however, also in case of the treatment with control hiPS-copGFP-EVs (Fig. 5C, left). With respect to cardiac ion channels, we detected significant upregulation of a sodium channel *SCN5A* after stimulation with hiPS-miR-199a-EVs in comparison to control (Fig. 5C, right). The expression of a potassium channel *KCND3* was strongly upregulated in cells treated with hiPS-EVs derived from all types of genetically engineered cells, including control hiPS-copGFP-EVs.

Next, we examined any spontaneous cardiomyogenic differentiation of CFs without the necessity of differentiation medium, but after incubation with EVs. We treated CFs with hiPS-miRs-EVs for 24 h, and 7 days later measured the levels of expression of selected genes. Our analysis revealed that incubation with hiPS-miR-199a-EVs in particular significantly increased transcript levels of several genes involved in cardiomyogenesis, including *TNTC, MEF2C and MYCHCB* in CFs (Fig. 5C, left), as well as for sodium and potassium channels (*SCN5A* and *KCNJ2*) (Fig. 5C, right). Moreover, we detected the highest level for GATA4 protein in CFs treated with hiPS-miR-199a-EVs (Fig. 5D).

### hiPS-miR-1-EVs Reduce Apoptosis, Inflammatory Response and Fibrosis in CFs

**Fig. 6:**
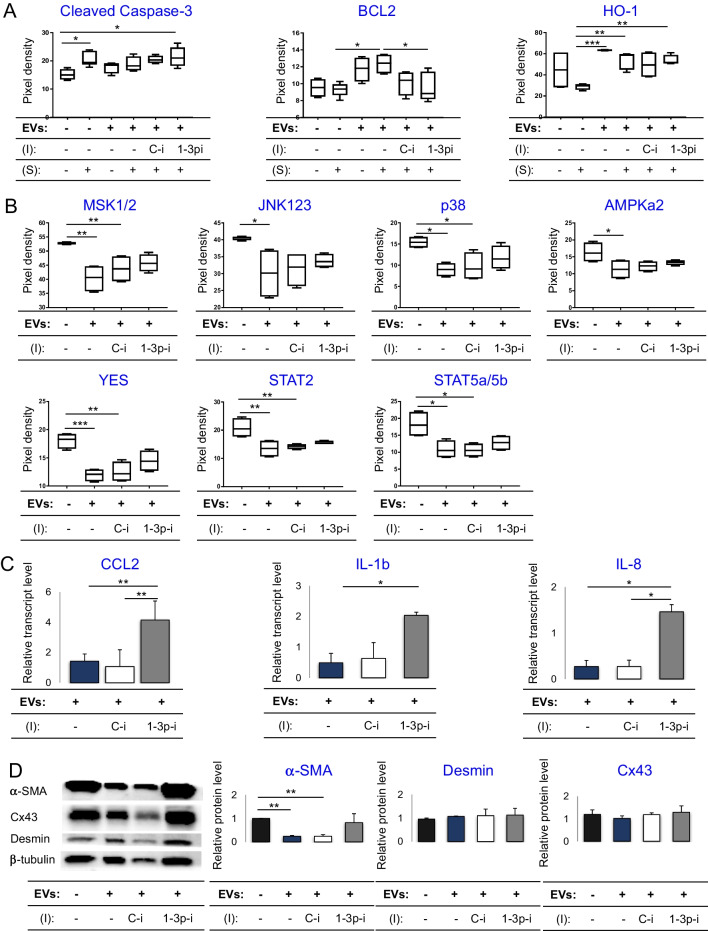
Activation of molecular pathways in CFs after treatment with hiPS-miR-1-EVs. (**A**) Protein array analysis of selected apoptosis- related proteins after incubation with EVs and cytotoxic agent (staurosporin; S), in the presence/absence of following miRNA inhibitors (I): specific miR-1-3p inhibitor (1-3p-i) or control inhibitor (C-i). (**B**) Levels of activated (phosphorylated) kinases in CFs treated with EVs and effects of respective inhibitors on protein levels. (**C**) Real-time qPCR analysis of expression of selected proinflammatory genes after treatment with EVs and with/ without respective inhibitors. The data were normalized compared with control untreated CFs. (**D**) Protein array analysis of fibrosis related proteins in CFs treated with EVs and with/ without respective inhibitor. ANOVA multiple comparisons and Tukey test; *p < 0.05, **p < 0.01

Having detected the anti-apoptotic properties of hiPS-miR-1-EVs on CFs, we examined the underlying molecular mechanisms and potential target genes responsible for miR-1-EV-mediated effects. Accordingly, we introduced miR-1-3p specific inhibitor to CFs after co-incubation with hiPS-miR-1-EVs and performed molecular analyses. First, CFs were treated with staurosporine and expression levels of various proteins involved in regulation of apoptosis were measured semi-quantitatively using the “Human Apoptosis Array Kit”. hiPS-miR-1-EVs reduced level of cleaved caspase-3 in CFs by upregulation of protective proteins, such BCL2 and HO-1 (Fig. 6A). Interestingly, hiPS-miR-1-EVs markedly reduced heat shock protein levels in CFs, particularly HSP60 (Supplementary Fig. 7), suggesting that the abundance of pro-survival signals played a predominant role in protecting CFs from apoptosis. To further investigate hiPS-miR-1-EVs function in CFs, we performed a proteome profiling of kinases activities in target CFs. In agreement with the above-described anti-proliferative effect of hiPS-miR-1-EVs on CFs (Fig. 4A), we detected significant downregulation of phosphorylated kinases: Mitogen- And Stress-Activated Protein Kinase 1/2 (MSK1/2), YES proto-oncogene 1, p38 Mitogen Activated Protein Kinase, Signal Transducer and Activator of Transcription (STAT)5a/5b, STAT2, AMP-activated Protein Kinase (AMPKa2), c-Jun N-Terminal Protein Kinase (JNK1/2/3) (Fig. 6B).

Next, to identify target genes for miR-1-3p in CFs, we selected 47 candidate genes using the TargetScan 7.1 database [33]. Our screen revealed a chemokine (C–C motif) ligand 2 (CCL2) also known as monocyte chemoattractant protein 1 (MCP1) to be targeted by miR-1 in CFs (Fig. 6C and Supplementary Table 3). Since CCL2 plays pro-inflammatory role, we wanted to verify whether its inhibition by hiPS-miR-1-EVs results in downregulation of other pro-inflammatory molecules released by CFs. Indeed, we found significant decrease in transcript levels of interleukin 1b (IL-1b) and interleukin 8 (IL-8) upon hiPS-miR-1-EVs treatment in CFs (Fig. 6C). The inhibitory effect was abrogated after addition of miR-1 inhibitor. Although other important fibrosis related genes such as collagen may play important role in this process, our search did not identified this genes as targets for miR-1. Knowing that pro-inflammatory properties of CFs are linked to development of fibrosis, we analyzed levels of pro-fibrotic proteins in CFs. Notably, high expression of alpha smooth muscle actin (α-SMA), which is a hallmark of myofibroblasts [34], was substantially decreased after CFs treatment with hiPS-miR-1-EVs (Fig. 6D). Again, addition of miR-1 inhibitor led to reduced expression on both protein (Fig. 6D) and mRNA (not shown) levels. However, expression of other pro-fibrotic proteins, such as connexin 43 (Cx43) and desmin, were unaffected by hiPS-miR-1-EVs treatment (Fig. 6D).

### hiPS-miR199a-EVs Induce Cardiomyogenesis and Favourably Modulates Pro-inflammatory and Pro-fibrotic Genes in CFs

**Fig. 7:**
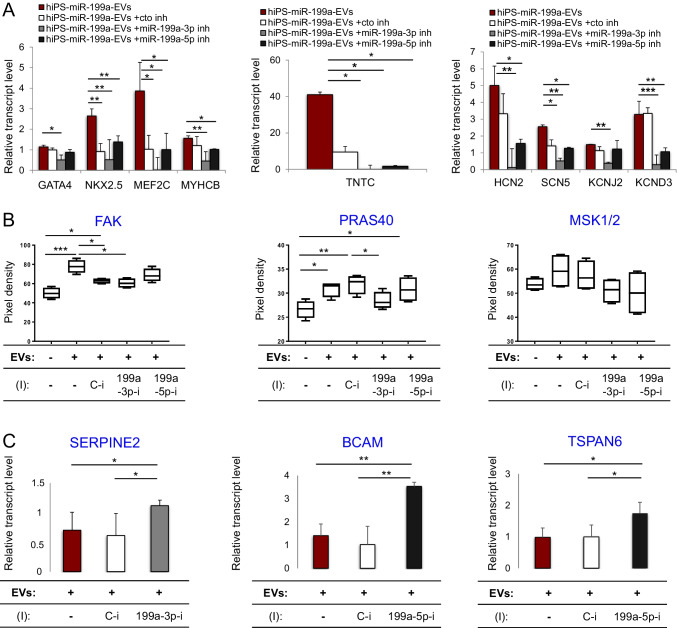
Activation of molecular pathways in CFs after treatment with hiPS-miR-199a-EVs. (**A**) RT-qPCR analysis of cardiomyogenic and cardiac ion channels-related genes in CFs treated with EVs and with/ without inhibitors (I): specific for miR-199a-3p (1991-3p-i), specific for miR-199a-5p (199a-5p-i) or control inhibitor (C-i). (**B**) Protein array analysis of activated kinases in CFs treated with EVs and with respective inhibitors. (**C**) Results of target scan analysis in CFs searching for candidate genes impacted by miR-199a-3p or miR-199a-5p, using RT-qPCR method. The data were normalized compared with control untreated CFs. ANOVA multiple comparisons and Tukey test; *p < 0.05, **p < 0.01

After we observed the cardiomyogenic effects of hiPS-miR-199a-EVs on CFs (Fig. 5C, D), we investigated which miR-199a variant (3p or 5p) was responsible for this effect. Thus, we treated CFs with hiPS-miR-199a-EVs and subsequently with inhibitor for miR-199a-3p or miR-199a-5p. Thereafter, CFs were cultured for 7 days in serum-reduced medium to induce spontaneous differentiation. Next, we analyzed transcript levels of genes related to cardiomyogenesis and encoding ion channels regulating cell contractility. The addition of miR-199a-3p inhibitor led to significant downregulation in expression of genes belonging to the cardiomyogenic differentiation pathway,including *GATA4, NKX2.5, MEF2C, TNTC*, and *MYHCB*, as well as for the genes encoding ion channels (Fig. 7A). These results suggest a regulatory role of miR-199a-3p in cardiac lineage specification in CFs. Next, we wanted, to define which intracellular pathways were affected by hiPS-miR-199a-EVs in CFs. Therefore, we measured protein levels of selected kinases in CFs. We detected elevated levels of phosphorylated Focal Adhesion Kinase (FAK), Proline-Rich Akt Substrate, 40 KDa (PRAS40) and MSK1/2 after treatment with hiPS-miR-199a-EVs (Fig. 7B). Decreased levels of these kinases were detected after addition of hiPS-miR-199a-3p inhibitor to CFs, indicating an important regulatory role of miR-199-3p in modulation of CF function. Going a step further, we performed a screen of target genes in CFs upon hiPS-miR-199a-EVs treatment and addition of respective inhibitors. Among selected candidate genes, we found *SERPINE2* to be specifically targeted by miR-199a-3p in CFs (Fig. 7C and Supplementary Table 4) and two target genes: *BCAM* and *TSPAN6* for miR-199a-5p (Fig. 7C and Supplementary Table 5), which are linked to inflammation and fibrosis.

## Discussion

Cardiovascular diseases continue to account for significant morbidity and mortality throughout the world. Due to very limited regenerative capacity of the human heart and failure of previous cell-based attempts to reconstitute damaged heart tissue, the need for novel treatment options is warranted. Because CFs represent a major cell population responsible for heart dysfunction due to the induction of fibrosis, we aimed to modify their biological behavior with miRNA-enriched-EVs to prevent adverse heart tissue remodeling.

Growing body of evidence indicates important regulatory role of miRNAs not only in CMs, but explicitly in CFs [35, 36] highlighting the relevance of impacting biological properties of the CFs by treatment with selected miRNAs. However, whether stem cells-derived EVs as carriers of miR-1 and miR-199a can attenuate inflammatory and pro-fibrotic role of CFs and induce cardiomyogenesis in CFs has not been examined. Therefore, we utilized EVs from genetically modified hiPS UC-MSC overexpressing miR-1 or miR-199a, to further boost positive outcome of stem cell-EV-based therapy.

Importantly, as shown by Agarwal et al. in their elegant study, CFs constitute the major target cell population for the uptake of small EVs (exosomes) among cells that reside within the myocardium [37]. The highest concentration of fluorescently-labeled exosomes was identified in fibroblasts, followed by cardiac endothelial cells and the lowest fluorescence intensity was detected in CMs. Moreover, although low amount of exosomes was transferred into CMs, this did not trigger any substantial functional effect in the recipient cells.

These data support our notion of that targeting CFs with miRs-enriched EVs may produce superior outcomes in future EV-based therapy for heart diseases.

We used stem cells-derived EVs enriched with miR-1 and miR-199a, whose proven functional effects may be beneficial for attenuating fibrosis and enhancing cardiomyogenesis [18–20, 24, 25].

We performed comprehensive functional and molecular studies to decipher the mechanisms of the miR-1/miR-199a-EVs effects on CF biological functions. Our results show the effects of hiPS-miR-1/miR-199a-EVs toward ameliorating inflammatory and pro-fibrotic properties of CFs and triggering cardiomyogenesis. These data may further be advanced for the development of novel cardiovascular therapies.

In particular, we discovered anti-proliferative and cytoprotective role of hiPS-miR-1-EVs on CFs, which may be important in prevention of excessive cell death upon ischemia–reperfusion and amelioration of fibrosis. Treatment of CFs with hiPS-miR-1-EVs substantially increased intracellular levels of pro-survival proteins Bcl-2 and HO-1. The role of HO-1 in cardioprotection after ischemia–reperfusion has been well documented [38]. HO-1 regulates inflammatory signaling and mitochondrial function, ultimately leading to heart tissue recovery upon injury. Similarly, overexpression of Bcl-2 significantly reduced ischemia–reperfusion-induced heart damage in an animal model, by modulating energy metabolism and mitigating acidification [39, 40]. Collectively, we reveal a novel role of miR-1-EVs in CFs, which seems to be opposite to miR-1 function in CMs. It has been shown that miR-1 targets cl in CMs directing cells towards apoptosis [41, 42]. However, miR-1 delivery to the mouse hearts attenuated pathological hypertrophy and fibrosis [17–20]. Similarly, restoration of physiological miR-1 levels in the heart by transgenic delivery of SERCA2a improved cardiac function [43]. These data suggest that careful optimization of the correct timing of the administration of miR-1-EVs would be prerequisite for achieving optimal results of their proregenerative activity.

Importantly, by identification of *CCL2* gene as a target for miR-1 in CFs, we revealed the anti-inflammatory function of hiPS-miR-1-EVs. Since the CCL2 protein is an important mediator of inflammatory pathways in CFs, which ultimately contribute to heart failure [44, 45], decreasing CCL2 levels may inhibit pathological inflammatory cascade in the post-ischemic heart. Indeed, transcript levels of other pro-inflammatory cytokines, including IL-1β and IL-8 were also reduced in CFs after treatment with hiPS-miR-1-EVs. Furthermore, knowing that inflammation is a stage preceding the development of fibrosis [6, 7], we evaluated expression levels of pro-fibrotic proteins in CFs. We detected significant downregulation of α-SMA expression in CFs, after treatment with hiPS-miR-1-EVs. The inhibitory effect was reverted upon CFs treatment with miR-1-3p inhibitor, indicating a causal role of miR-1 in this process. Together, these data indicate that hiPS-miR-1-EVs induce cytoprotective, anti-inflammatory and anti-fibrotic attributes in CFs.

With respect to hiPS-mir-199a-EVs, we demonstrated their cardiomyogenic function in CFs. By inducing the expression of a number of genes involved in cardiomyogenesis, such as *NKX2.5, TNTC, MEF2C* and *MYCHCB*, and the ion channels necessary for CM contractility, hiPS-miR-199a-EVs may offer the advantage of epigenetic conversion of CFs to CMs, or at least enhancing CFs properties to reconstitute functional heart tissue. Importantly, miRNAs are able to mediate reprograming of CFs to CMs both *in vitro* and *in vivo* [36, 37]. Our studies with miR-199a specific inhibitors indicated a causal role of miR-199a-3p in inducing pro-cardiomyogenic effects in CFs. To delineate the underlying molecular mechanisms, we performed screening of kinases activities in CFs after hiPS-miR-199a-EVs treatment. We observed significantly enhanced phosphorylation of FAK and PRAS40. Since FAK has been implicated in key intracellular processes including heart development [46], we propose its regulatory role in driving cardiomyogenesis in CFs. In turn, PRAS40 activation in CFs after treatment with hiPS-miR-199a-EVs points towards anti-fibrotic and anti-inflammatory function. Notably, mice over-expressing PRAS40 exhibited attenuated fibrotic remodeling and decreased hypertrophy [47].

To further decipher components of molecular effects of miR-199a-expessing EVs in CFs, we screened for target genes in recipient cells. Our data indicated *SERPINE2* as a target for miR-199a-3p in CFs and *BCAM*, as well as *TSPAN6* for miR-199a-5p. *SERPINE* has been implicated in the development of myocardial fibrosis via collagen deposition [48, 49], thus downregulating its expression may lead to reduction of scar tissue and improved heart function. *BCAM* and *TSPAN6* are involved in signal transduction. Importantly, BCAM was recently identified as target of miR-199a-5p in skin keratinocytes, where it acts as a tumor suppressor by inhibiting invasiveness [50], and in placenta, where it inhibits trophoblast proliferation, migration and invasion [51]. These activities of *BCAM* may support the safety of our approach of hiPS-miRs-EVs delivery to the heart in future therapy. Function of tetraspanins has not been studied in CFs, although their role in regulation of inflammation was depicted in endothelial cells [52]. Therefore, by dowregulating their target genes, miR-199a-3p and miR-199a-5p may suppress inflammation and fibrosis and enhance cardiomyogenesis in CFs. Importantly, in light of a recent report, conversion of fibroblasts to CMs can substantially be enhanced by inhibiting pro-fibrotic signaling [53]. Thus, in our model, the observed cardiomyogeneic effect of hiPS-miR-199a-EVs may be supported by the inhibition of pro-fibrotic signaling. However, further studies are necessary to fully understand the mechanisms behind hiPS-miR-199a-EVs-medited induction of cardiomyogenesis.

Another advantage of our approach is the use of producer cells stably overexpressing specific miRNAs for EV collection. Thus, we overcome the need for repeated EVs transfection with costly synthetic miRNAs, an aspect important for the future good manufacturing practice (GMP)-based preparation of transplantable biological products. Moreover, our discovery that upregulation of one miRNA can entail elevated levels of other miRNAs confirms that a fine-tuned miRNA regulatory network exists in cells. Thus, it is likely that the cellular response not to a single miRNA but to different interacting miRNAs that determines the ultimate effects. The identified pathways for miR-199a-EVs implicate TGFβ, FOXO, and Hippo signaling and regulation of pluripotency, which were have been shown to be important for heart function and recovery [54–56]. These provide additional support toward feasibility and efficacy of miRNA-EVs-based therapies for heart repair.

## Conclusions

Our data indicate that hiPS-miR-1-EV treatment protects CFs from apoptosis, decreases their proliferation and therefore, may mitigate the risk of fibrosis in the injured heart. hiPS-miR-1-EV treatment of CFs also attenuates inflammatory reaction by targeting CCL-2 and reducing IL-1β and IL-8. Further, by regulating the production of pro-fibrotic proteins such as α-SMA, hiPS-miR-1EVs may reduce the scar formation and ameliorate stiffness of the infarct zone. Treatment with hiPS-miR-199a-EVs induces cardiomyogenesis and increases expression of transcripts encoding ion channels, enabling superior signal transduction and enhanced cardiomyocyte contractility. By targeting SERPINE2, BCAM and TSPAN6, miR-199a-EVs may further reduce inflammation and fibrosis, bringing synergistic reparative effects. Thus, targeting CFs with specific miR-EVs may create an environment that would favor myocardial repair and reconstitution.

### Supplementary Information

Below is the link to the electronic supplementary material.

Supplementary file1 (PDF 954 KB)

## Data Availability

The data and materials generated during this study are available from the corresponding author upon request to any qualified researcher.

## Abbreviations

EVs: Extracellular vesicles
CFs: Cardiac fibroblasts
hiPS: Human induced pluripotent stem cells
UC-MSCs: Umbilical cord-derived mesenchymal stem cells
hiPS-EVs: Human induced pluripotent stem cell–derived extracellular vesicles
UC-MSC-EVs: Umbilical cord-derived mesenchymal stem cells–derived extracellular vesicles
miR-EVs: MicroRNA-enriched extracellular vesicles
CMs: Cardiomyocytes
MI: Myocardial infarction
WT: Wild type
FMT: Fibroblast-to-myofibroblast transition

## Code Availability

N/A

## References

[1] Roth GA, Mensah GA, Johnson CO, Addolorato G, Ammirati E, Baddour LM, Barengo NC, Beaton AZ, Benjamin EJ, Benziger CP (2020). Global burden of cardiovascular diseases and risk factors, 1990–2019: Update from the GBD 2019 study. Journal of the American College of Cardiology.

[2] Hashimoto H, Olson EN, Bassel-Duby R (2018). Therapeutic approaches for cardiac regeneration and repair. Nature Reviews Cardiology.

[3] Li X, Tamama K, Xie X, Guan J (2016). Improving cell engraftment in cardiac stem cell therapy. Stem Cells International.

[4] Ong SB, Hernandez-Resendiz S, Crespo-Avilan GE, Mukhametshina RT, Kwek XY, Cabrera-Fuentes HA, Hausenloy DJ (2018). Inflammation following acute myocardial infarction: Multiple players, dynamic roles, and novel therapeutic opportunities. Pharmacology & Therapeutics.

[5] Travers JG, Kamal FA, Robbins J, Yutzey KE, Blaxall BC (2016). Cardiac fibrosis: The fibroblast awakens. Circulation Research.

[6] Prabhu SD, Frangogiannis NG (2016). The biological basis for cardiac repair after myocardial infarction: From inflammation to fibrosis. Circulation Research.

[7] Jiang W, Xiong Y, Li X, Yang Y (2021). Cardiac fibrosis: Cellular effectors, molecular pathways, and exosomal roles. Frontiers in Cardiovascular Medicine.

[8] Femmino S, Bonelli F, Brizzi MF (2022). Extracellular vesicles in cardiac repair and regeneration: Beyond stem-cell-based approaches. Frontiers in Cell and Developmental Biology.

[9] de Abreu RC, Fernandes H, da Costa Martins PA, Sahoo S, Emanueli C, Ferreira L (2020). Native and bioengineered extracellular vesicles for cardiovascular therapeutics. Nature Reviews Cardiology.

[10] Cheng L, Hill AF (2022). Therapeutically harnessing extracellular vesicles. Nature Reviews Drug Discovery.

[11] Kim HY, Kwon S, Um W, Shin S, Kim CH, Park JH, Kim BS (2022). Functional extracellular vesicles for regenerative medicine. Small.

[12] Karnas E, Sekula-Stryjewska M, Kmiotek-Wasylewska K, Bobis-Wozowicz S, Ryszawy D, Sarna M, Madeja Z, Zuba-Surma EK (2021). Extracellular vesicles from human iPSCs enhance reconstitution capacity of cord blood-derived hematopoietic stem and progenitor cells. Leukemia.

[13] Adamiak M, Cheng G, Bobis-Wozowicz S, Zhao L, Kedracka-Krok S, Samanta A, Karnas E, Xuan YT, Skupien-Rabian B, Chen X (2018). Induced Pluripotent Stem Cell (iPSC)-derived extracellular vesicles are safer and more effective for cardiac repair than iPSCs. Circulation Research.

[14] Wang H, Xie Y, Guan L, Elkin K, Xiao J (2021). Targets identified from exercised heart: Killing multiple birds with one stone. npj Regenerative Medicine.

[15] Song Y, Zhang C, Zhang J, Jiao Z, Dong N, Wang G, Wang Z, Wang L (2019). Localized injection of miRNA-21-enriched extracellular vesicles effectively restores cardiac function after myocardial infarction. Theranostics.

[16] Pan Y, Liu Y, Wei W, Yang X, Wang Z, Xin W (2023). Extracellular vesicles as delivery shippers for noncoding RNA-based modulation of angiogenesis: insights from ischemic stroke and cancer. Small.

[17] Sadat-Ebrahimi SR, Rezabakhsh A, Aslanabadi N, Asadi M, Zafari V, Shanebandi D, Zarredar H, Enamzadeh E, Taghizadeh H, Badalzadeh R (2022). Novel diagnostic potential of miR-1 in patients with acute heart failure. PLoS One.

[18] Valkov N, King ME, Moeller J, Liu H, Li X, Zhang P (2019). MicroRNA-1-mediated inhibition of cardiac fibroblast proliferation through targeting cyclin D2 and CDK6. Frontiers in Cardiovascular Medicine.

[19] Yin H, Zhao L, Zhang S, Zhang Y, Lei S (2015). MicroRNA-1 suppresses cardiac hypertrophy by targeting nuclear factor of activated T cells cytoplasmic 3. Molecular Medicine Reports.

[20] Karakikes I, Chaanine AH, Kang S, Mukete BN, Jeong D, Zhang S, Hajjar RJ, Lebeche D (2013). Therapeutic cardiac-targeted delivery of miR-1 reverses pressure overload-induced cardiac hypertrophy and attenuates pathological remodeling. Journal of the American Heart Association.

[21] Chen X, Gu S, Chen BF, Shen WL, Yin Z, Xu GW, Hu JJ, Zhu T, Li G, Wan C (2015). Nanoparticle delivery of stable miR-199a-5p agomir improves the osteogenesis of human mesenchymal stem cells via the HIF1a pathway. Biomaterials.

[22] Hou Y, Fu L, Li J, Li J, Zhao Y, Luan Y, Liu A, Liu H, Li X, Zhao S (2018). Transcriptome analysis of potential miRNA involved in adipogenic differentiation of C2C12 myoblasts. Lipids.

[23] Cianflone E, Scalise M, Marino F, Salerno L, Salerno N, Urbanek K, Torella D (2022). The negative regulation of gene expression by microRNAs as key driver of inducers and repressors of cardiomyocyte differentiation. Clinical Science (London).

[24] Lesizza P, Prosdocimo G, Martinelli V, Sinagra G, Zacchigna S, Giacca M (2017). Single-dose intracardiac injection of pro-regenerative MicroRNAs improves cardiac function after myocardial infarction. Circulation Research.

[25] Gabisonia K, Prosdocimo G, Aquaro GD, Carlucci L, Zentilin L, Secco I, Ali H, Braga L, Gorgodze N, Bernini F (2019). MicroRNA therapy stimulates uncontrolled cardiac repair after myocardial infarction in pigs. Nature.

[26] Bobis-Wozowicz S, Kmiotek K, Kania K, Karnas E, Labedz-Maslowska A, Sekula M, Kedracka-Krok S, Kolcz J, Boruczkowski D, Madeja Z (2017). Diverse impact of xeno-free conditions on biological and regenerative properties of hUC-MSCs and their extracellular vesicles. Journal of Molecular Medicine (Berlin).

[27] Bobis-Wozowicz S, Kmiotek K, Sekula M, Kedracka-Krok S, Kamycka E, Adamiak M, Jankowska U, Madetko-Talowska A, Sarna M, Bik-Multanowski M (2015). Human induced pluripotent stem cell-derived microvesicles transmit RNAs and proteins to recipient mature heart cells modulating cell fate and behavior. Stem Cells.

[28] Garikipati VNS, Shoja-Taheri F, Davis ME, Kishore R (2018). Extracellular vesicles and the application of system biology and computational modeling in cardiac repair. Circulation Research.

[29] Thery C, Witwer KW, Aikawa E, Alcaraz MJ, Anderson JD, Andriantsitohaina R, Antoniou A, Arab T, Archer F, Atkin-Smith GK (2018). Minimal information for studies of extracellular vesicles 2018 (MISEV2018): A position statement of the International Society for Extracellular Vesicles and update of the MISEV2014 guidelines. Journal of Extracellular Vesicles.

[30] Vlachos IS, Zagganas K, Paraskevopoulou MD, Georgakilas G, Karagkouni D, Vergoulis T, Dalamagas T, Hatzigeorgiou AG (2015). DIANA-miRPath v3.0: deciphering microRNA function with experimental support. Nucleic Acids Research.

[31] Liu J, Wang Y, Ji P, Jin X (2020). Application of the microRNA-302/367 cluster in cancer therapy. Cancer Science.

[32] Nguyen PNN, Choo KB, Huang CJ, Sugii S, Cheong SK, Kamarul T (2017). miR-524-5p of the primate-specific C19MC miRNA cluster targets TP53IPN1- and EMT-associated genes to regulate cellular reprogramming. Stem Cell Research & Therapy.

[33] Agarwal, V., Bell, G. W., Nam, J. W., & Bartel, D. P. (2015). Predicting effective microRNA target sites in mammalian mRNAs. *Elife, 4*. 10.7554/eLife.05005 10.7554/eLife.05005 PMC4532895 26267216

[34] Liu M, de Juan L, Abad B, Cheng K (2021). Cardiac fibrosis: Myofibroblast-mediated pathological regulation and drug delivery strategies. Advanced Drug Delivery Reviews.

[35] Yang K, Shi J, Hu Z, Hu X (2019). The deficiency of miR-214-3p exacerbates cardiac fibrosis via miR-214-3p/NLRC5 axis. Clinical Science (London).

[36] Paoletti C, Divieto C, Tarricone G, Di Meglio F, Nurzynska D, Chiono V (2020). MicroRNA-mediated direct reprogramming of human adult fibroblasts toward cardiac phenotype. Frontiers in Bioengineering and Biotechnology.

[37] Agarwal U, George A, Bhutani S, Ghosh-Choudhary S, Maxwell JT, Brown ME, Mehta Y, Platt MO, Liang Y, Sahoo S (2017). Experimental, systems, and computational approaches to understanding the MicroRNA-mediated reparative potential of cardiac progenitor cell-derived exosomes from pediatric patients. Circulation Research.

[38] Tift MS, Alves de Souza RW, Weber J, Heinrich EC, Villafuerte FC, Malhotra A, Otterbein LE, Simonson TS (2020). Adaptive potential of the heme oxygenase/carbon monoxide pathway during hypoxia. Frontiers in Physiology.

[39] Imahashi K, Schneider MD, Steenbergen C, Murphy E (2004). Transgenic expression of Bcl-2 modulates energy metabolism, prevents cytosolic acidification during ischemia, and reduces ischemia/reperfusion injury. Circulation Research.

[40] Tanaka M, Nakae S, Terry RD, Mokhtari GK, Gunawan F, Balsam LB, Kaneda H, Kofidis T, Tsao PS, Robbins RC (2004). Cardiomyocyte-specific Bcl-2 overexpression attenuates ischemia-reperfusion injury, immune response during acute rejection, and graft coronary artery disease. Blood.

[41] Tang Y, Zheng J, Sun Y, Wu Z, Liu Z, Huang G (2009). MicroRNA-1 regulates cardiomyocyte apoptosis by targeting Bcl-2. International Heart Journal.

[42] Zhai C, Tang G, Peng L, Hu H, Qian G, Wang S, Yao J, Zhang X, Fang Y, Yang S (2015). Inhibition of microRNA-1 attenuates hypoxia/re-oxygenation-induced apoptosis of cardiomyocytes by directly targeting Bcl-2 but not GADD45Beta. American Journal of Translational Research.

[43] Kumarswamy R, Lyon AR, Volkmann I, Mills AM, Bretthauer J, Pahuja A, Geers-Knorr C, Kraft T, Hajjar RJ, Macleod KT (2012). SERCA2a gene therapy restores microRNA-1 expression in heart failure via an Akt/FoxO3A-dependent pathway. European Heart Journal.

[44] Lindner D, Zietsch C, Tank J, Sossalla S, Fluschnik N, Hinrichs S, Maier L, Poller W, Blankenberg S, Schultheiss HP (2014). Cardiac fibroblasts support cardiac inflammation in heart failure. Basic Research in Cardiology.

[45] Frangogiannis NG, Dewald O, Xia Y, Ren G, Haudek S, Leucker T, Kraemer D, Taffet G, Rollins BJ, Entman ML (2007). Critical role of monocyte chemoattractant protein-1/CC chemokine ligand 2 in the pathogenesis of ischemic cardiomyopathy. Circulation.

[46] Doherty JT, Conlon FL, Mack CP, Taylor JM (2010). Focal adhesion kinase is essential for cardiac looping and multichamber heart formation. Genesis.

[47] Volkers M, Toko H, Doroudgar S, Din S, Quijada P, Joyo AY, Ornelas L, Joyo E, Thuerauf DJ, Konstandin MH (2013). Pathological hypertrophy amelioration by PRAS40-mediated inhibition of mTORC1. Proceedings of the National Academy of Sciences (PNAS).

[48] Li X, Zhao D, Guo Z, Li T, Qili M, Xu B, Qian M, Liang H, X E, ChegeGitau S (2016). Overexpression of SerpinE2/protease nexin-1 contribute to pathological cardiac fibrosis via increasing collagen deposition. Scientific Reports.

[49] Li C, Lv LF, Qi-Li MG, Yang R, Wang YJ, Chen SS, Zhang MX, Li TY, Yu T, Zhou YH (2022). Endocytosis of peptidase inhibitor SerpinE2 promotes myocardial fibrosis through activating ERK1/2 and beta-catenin signaling pathways. International Journal of Biological Sciences.

[50] Kim BK, Kim I, Yoon SK (2015). Identification of miR-199a-5p target genes in the skin keratinocyte and their expression in cutaneous squamous cell carcinoma. Journal of Dermatological Science.

[51] Liu M, Liao L, Gao Y, Yin Y, Wei X, Xu Q, Gao L, Zhou R (2022). BCAM Deficiency may contribute to preeclampsia by suppressing the PIK3R6/p-STAT3 signaling. Hypertension.

[52] Yeung L, Hickey MJ, Wright MD (2018). The many and varied roles of tetraspanins in immune cell recruitment and migration. Frontiers in Immunology.

[53] Zhao Y, Londono P, Cao Y, Sharpe EJ, Proenza C, O'Rourke R, Jones KL, Jeong MY, Walker LA, Buttrick PM (2015). High-efficiency reprogramming of fibroblasts into cardiomyocytes requires suppression of pro-fibrotic signalling. Nature Communications.

[54] Norambuena-Soto I, Nunez-Soto C, Sanhueza-Olivares F, Cancino-Arenas N, Mondaca-Ruff D, Vivar R, Diaz-Araya G, Mellado R, Chiong M (2017). Transforming growth factor-beta and Forkhead box O transcription factors as cardiac fibroblast regulators. BioScience Trends.

[55] Diez-Cunado M, Wei K, Bushway PJ, Maurya MR, Perera R, Subramaniam S, Ruiz-Lozano P, Mercola M (2018). miRNAs that induce human cardiomyocyte proliferation converge on the hippo pathway. Cell Reports.

[56] Torrini C, Cubero RJ, Dirkx E, Braga L, Ali H, Prosdocimo G, Gutierrez MI, Collesi C, Licastro D, Zentilin L (2019). Common regulatory pathways mediate activity of MicroRNAs inducing cardiomyocyte proliferation. Cell Reports.

