# Supplementary material for: Anti-inflammatory, Anti-fibrotic and Pro-cardiomyogenic Effects of Genetically Engineered Extracellular Vesicles Enriched in miR-1 and miR-199a on Human Cardiac Fibroblasts
Source: Stem Cell Rev Rep. 2023 Sep 13;19(8):2756–73. doi: 10.1007/s12015-023-10621-2 (PMC10661813; doi:10.1007/s12015-023-10621-2)
Supplement: Supplementary file 1 — Supplementary file1 (PDF 954 KB) [file 12015_2023_10621_MOESM1_ESM.pdf]

SUPPLEMENTARY INFORMATION

Anti-inflammatory, anti-fibrotic and pro-cardiomyogenic effects of genetically engineered extracellular vesicles enriched in miR-1 and miR-199a on human cardiac fibroblasts

Stem Cell Reviews and Reports

Katarzyna Kmiotek-Wasylewska[1#], Sylwia Bobis-Wozowicz[1#], Elżbieta Karnas[1], Monika Orpel[1], Olga Woznicka[2], Zbigniew Madeja[1], Buddhadeb Dawn[3] and Ewa Zuba-Surma[1]*

[1]Jagiellonian University, Faculty of Biochemistry, Biophysics and Biotechnology, Department of Cell Biology, Krakow, Poland

[2]Jagiellonian University, Institute of Zoology and Biomedical Research, Department of Cell Biology and Imaging, Kraków, Poland

[3]Department of Internal Medicine, Kirk Kerkorian School of Medicine at the University of Nevada, Las Vegas, Las Vegas, NV, USA

***E-mail address of the corresponding author:** ewa.zuba-surma@uj.edu.pl

SUPPLEMENTARY METHODS

Time-lapse monitoring of cells migratory activity

CFs s were seeded into 12-well plates, at a density of 20 000 cells per well. Cells were treated with EVs (20 ng/1000 cells) for 1h and migratory activity was next recorded for 12h at 10-minute intervals using Leica DMI6000B inverted microscope equipped with a dry 10x, NA 0.25 objective and a digital DFC360FX CCD camera (Leica Microsystems) and incubator chamber (37°C and 5% CO_2). Cell trajectories were constructed from a sequence of cell centroid positions, pooled and analyzed with the Hiro program (written by W. Czapla). Two hundred (200) different cells per each sample were analyzed. Cell trajectories, from three independent experiments, were taken for the statistical analysis in order to obtain the average speed of cell movement (μm/h) and total length of cell displacement (μm).

SUPPLEMENTARY TABLES

Supplementary Table 1. List of primer sequences used in RT-qPCR.

Gene name	Sequence	
GATA4	F	AACGACGGCAACAACGATAAT
	R	GTTTTTTCCCCTTTGATTTTTGATC
NKX2.5	F	CCCCTGGATTTTGCATTCAC
	R	CGTGCGCAAGAACAAACG
TNTC	F	ATGAGCGGGAGAAGGAGCGGCAGAAC
	R	TCAATGGCCAGCACCTTCCTCCTCTC
MEF2C	F	TTTAACACCGCCAGCGCTCTTCACCTTG
	R	TCGTGGCGCGTGTGTTGTGGGTATCTCG
MYCHCB	F	CTGGAGGCCGAGCAGAAGCGCAACG
	R	GTCCGCCCGCTCCTCTGCCTCATCC
HCN2	F	CCAGCTGTAAGACAGGGACG
	R	GCGGGCCAAGTATTGCACTT
SCN5A	F	CCAGATCTCTATGGCAATCCA
	R	GAATCTTCACAGCCGCTCTC
KCNJ2	F	TACGAAGTCCCCAACACTCC
	R	CTTTGCTTGTGAGGGCAACT
KCND3	F	GTTTGAGCAGAACTGCATGG
	R	GTGGATCGTGCTGAGCTCTT
IL-1b	F	AGACATCACCAAGCTTTTTTGCT
	R	GCACGATGCACCTGTACGAT
IL-8	F	TTAGCACTCCTTGGCAAAACTG
	R	CTGGCCGTGGCTCTCTTG
α-SMA	F	CTGTTCCAGCCATCCTTCAT
	R	CCGTGATCTCCTTCTGCATT
BAX	F	CATGGGCTGGACATTGGACT

	R	AAAGTAGGAGAGGAGGCCGT
BCL2	F	GAGTGACAGTGGATTGCAT
	R	CAGAATATCAGCCACCTCTT
c-MYC	F	TCTCCGTCCTCGGATTCTCT
	R	TTCTTGTTCCTCCTCAGAGTCG
p21	F	CTCAGGGTCGAAAACGGCGG
	R	CAGGCTTCCTGTGGGCGGAT
CDK4	F	CCTCTCTAGCTTGCGGCCT
	R	CAGATCAAGGGAGACCCTCACG
CCND1	F	ATGCCAACCTCCTCAACGAC
	R	TCTGTTCCTCGCAGACCTCC
CDK2	F	GGCACGTACGGAGTTGTGT
	R	CCTCAGTCTCAGTGTCCAGGC
CCNE1	F	GCAGGATCCAGATGAAGAAATG
	R	TTTGCCCAGCTCAGTACAGG
b2M	F	AATGCGGCATCTTCAAACCT
	R	TGACTTTGTCACAGCCCAAGATA

Supplementary Table 2. Analysis of size and particle concentration in samples of hiPS-miRs-EVs and UC-MSC-miRs-EVs using NTA.

**EV type**	**Size analysis**		
	Mean +/- SE [nm]	Mode +/- SE [nm]	Concentration [no/ml]
hiPS-WT-EVs	133.1 +/- 0.3	87.9 +/- 2.8	1.98×10^{11} +/- 8.17×10^{9}
hiPS-copGFP-EVs	187.3 +/- 4.7	127.7 +/- 7.3	1.72×10^{11} +/- 4.68×10^{9}
hiPS-miR-1-EVs	213.2 +/- 2.1	134.9 +/- 3.4	2.81×10^{11} +/- 8.78×10^{9}
hiPS-miR-199a-EVs	167.1 +/- 4.1	107.6 +/- 11.7	3.23×10^{11} +/- 3.17×10^{9}
UC-MSC-WT-EVs	119.4 +/- 2.3	90.6 +/- 2.3	6.06×10^{11} +/- 3.70×10^{9}
UC-MSC-copGFP-EVs	113.6 +/- 3.2	91.5 +/- 6.4	3.16×10^{11} +/- 4.72×10^{9}
UC-MSC-miR-1-EVs	110.7 +/- 0.6	81.1 +/- 3.5	7.26×10^{11} +/- 3.71×10^{9}
UC-MSC-miR-199a-EVs	117.1 +/- 0.4	97.7 +/- 1.4	6.62×10^{11} +/- 1.81×10^{9}

Supplementary Table 3. Target genes identification in CFs treated with hiPS-EV-miR-1 and with/without miR-1-3p inhibitor (or control inhibitor; C-i). The 47 listed genes were highlighted as miR-1 targets by searching the TargetScan 7.1 database.

**Target gene**	**- (no inhibitor)**		**+C-i**		**+ miR-1-3p-i**	
	Mean	SD	Mean	SD	Mean	SD
ARPC3	1.12	0.25	1.03	0.15	1.00	0.11
SERP1	1.09	0.22	1.10	0.20	1.07	0.11
MAP3K13	1.70	0.48	1.63	0.23	1.39	0.15
**CCL2**	**1.43**	**0.47**	**1.08**	**1.10**	**4.15**	**1.26**
BDNF	1.30	0.55	0.67	0.19	1.08	0.45
CDK14	1.54	0.22	1.56	0.15	1.69	0.33
UST	2.17	0.61	1.95	0.99	2.14	0.64
SEPT2	4.46	0.97	4.18	0.69	3.97	1.72

KCNMB2	2.96	1.85	2.91	1.52	2.95	1.33
CCSAP	1.43	0.28	1.57	0.47	1.18	0.61
GSK3B	2.56	0.93	3.17	0.55	1.89	1.26
HNRNPU	6.18	1.15	5.16	2.48	4.32	1.81
TSPAN4	2.53	0.75	2.00	1.06	1.82	1.06
VAMP4	2.07	0.20	2.22	0.43	2.25	0.66
RGS7	nd	nd	nd	nd	nd	nd
CAAP1	1.68	0.28	1.64	0.21	1.60	0.32
PDCD10	1.04	0.37	0.58	0.04	0.64	0.21
GJA1	3.49	1.51	2.36	0.96	2.78	1.00
KIF2A	5.97	1.23	5.17	2.38	5.12	1.87
SLC44A1	2.41	0.58	2.25	0.92	2.45	1.02
SMAD2	1.14	0.25	1.08	0.13	1.13	0.13
PI3KP85	2.40	0.88	2.15	0.56	1.87	0.68
CLTC	5.83	0.93	5.38	0.20	4.44	1.85
CNN3	2.29	0.63	2.49	0.97	1.76	1.07
TIMP3	nd	nd	nd	nd	nd	nd
DDX5	4.04	0.80	3.12	1.13	2.92	1.14
FN1	6.53	1.32	4.83	0.22	5.76	2.51
CALM2	3.18	0.77	2.04	1.24	2.78	1.02
CALM1	4.31	1.44	4.69	0.48	3.65	1.51
TNPO1	2.30	1.12	2.30	0.93	2.03	0.80
CAPZA1	1.76	0.66	2.53	0.71	1.65	0.88
IGFBP5	5.30	0.95	5.50	4.36	6.33	3.13
CDK6	1.84	0.65	1.77	0.65	1.74	0.75
MYLK	nd	nd	nd	nd	nd	nd
TMX1	1.71	0.54	1.57	0.30	1.39	0.46
CCND1	4.92	2.22	9.04	2.85	3.07	2.75
SNAI2	1.05	0.21	1.08	0.18	1.09	0.56
ARCN1	3.16	1.11	3.93	1.12	2.80	1.63
TAGLN2	3.53	0.93	1.96	0.80	3.17	1.91
G6PD	3.42	1.03	3.83	0.12	3.02	2.18
HIPK1	5.48	0.71	4.29	2.17	5.82	2.71
IGF1	5.26	3.75	1.05	1.08	3.06	1.70
SMAD3	3.49	0.65	2.47	0.74	4.25	1.26
SRI	1.37	0.26	1.16	0.50	1.16	0.47
HDAC4	nd	nd	nd	nd	nd	nd
NOTCH3	2.04	1.22	1.39	0.52	3.08	0.81
DKK1	0.86	0.28	0.94	0.29	0.59	0.34

<u>Supplementary Table 4.</u> Target genes identification in CFs treated <u>with hiPS-EV-miR-199a</u> and with/without <u>miR-199a-3p inhibitor</u> (or control inhibitor; C-i).

Target gene	- (no inhibitor)		+C-i		+ miR199a-3p-i	
	Mean	SD	Mean	SD	Mean	SD
**SERPINE2**	**0.70**	**0.30**	**0.61**	**0.38**	**1.12**	**0.09**
PAWR	0.13	0.07	0.10	0.02	0.12	0.01
VAMP3	3.64	0.99	2.90	0.82	4.82	0.41
PAK4	0.01	0.00	0.01	0.00	0.01	0.00
CDK7	482.79	89.58	394.02	130.89	361.90	30.65
CYB5R4	0.14	0.04	0.13	0.04	0.18	0.05
MCFD2	17.73	5.44	20.50	1.66	24.61	2.08
CD151	13.25	0.44	10.91	0.73	9.36	0.79
MAP3K4	0.03	0.01	0.03	0.01	0.05	0.01
CELSR2	0.30	0.11	0.23	0.03	0.35	0.10
PTGS2	2866.82	850.68	3803.18	638.66	2046.99	173.36
CFL2	0.33	0.11	0.32	0.03	0.05	0.01
SCD	3.27	1.44	4.06	0.25	3.75	0.32
RNGTT	1.33	0.49	1.22	0.04	1.50	0.13
G3BP2	17.37	4.54	18.71	0.38	13.00	1.10
TFAM	0.89	0.36	0.79	0.18	1.24	0.11
BCL2L13	3.69	1.03	3.63	0.09	2.94	0.25
CD44	48.28	18.94	53.41	0.18	50.52	4.28
SUMO3	0.60	0.19	0.87	0.10	0.81	0.07
CALD1	18.25	5.73	15.01	0.05	8.58	0.73
OXSR1	0.11	0.04	0.10	0.01	0.10	0.01
SMAD1	1.25	0.22	1.17	0.13	0.77	0.07
ET1	29.30	14.66	24.72	0.93	20.05	1.70
HOMER1	0.11	0.04	0.11	0.00	0.07	0.01

<u>Supplementary Table 5.</u> Target genes identification in CFs treated <u>with hiPS-EV-miR-199a</u> and with/without <u>miR-199a-5p inhibitor</u> (or control inhibitor; C-i).

Target gene	- (no inhibitor)		+C-i		+ miR199a-5p-i	
	Mean	SD	Mean	SD	Mean	SD
ZNF776	2,58	0,92	1,89	1,07	2,51	0,27
**BCAM**	**1,41**	**0,50**	**1,02**	**0,79**	**3,54**	**0,18**
RAD23B	2,56	1,38	1,57	1,14	2,33	0,28
**TSPAN6**	**0,98**	**0,30**	**1,00**	**0,38**	**1,74**	**0,35**
ECE1	3,68	2,47	2,33	1,91	2,78	0,07
AKAP1	4,12	3,71	2,93	2,53	2,88	0,32
MAGT1	5,85	3,04	2,66	2,31	3,88	0,13
B3GNT1	1,00	0,63	0,81	0,48	1,18	0,10
ENO3	1,49	0,49	0,91	0,66	1,96	0,16
MAP3K11	2,03	1,50	1,55	1,22	2,23	0,00

HIF1A	2,25	1,27	1,45	0,97	1,11	0,11
ETS1	3,97	2,49	2,58	2,03	3,04	0,69
LAMC2	2,11	1,11	1,20	0,25	0,61	0,11
SET	1,17	0,77	1,02	0,44	1,25	0,05
CDKN1B	2,31	0,97	1,48	1,29	1,98	0,06
WNT2	1,68	1,26	0,25	0,12	0,22	0,03
GSK3B	1,94	0,93	1,21	0,90	1,57	0,03
CAV1	1,18	0,42	0,71	0,55	0,63	0,11
JAG1	2,22	2,14	3,63	1,14	1,35	0,18
FZD4	1,75	1,02	1,46	0,92	1,65	0,07
HSPA5	3,51	1,55	1,81	1,47	1,27	0,02
MGAT4B	2,78	1,50	1,88	1,59	2,39	0,14

SUPPLEMENTARY FIGURES

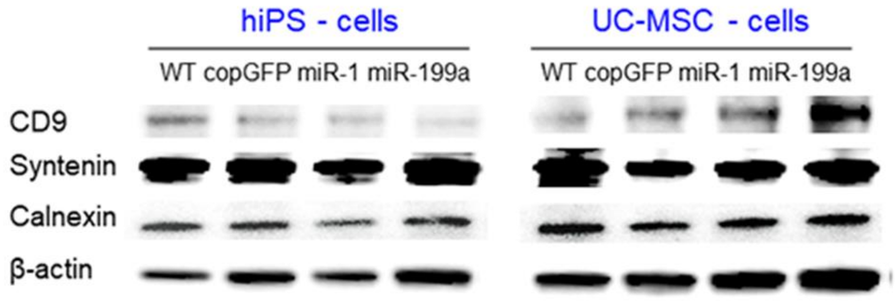

Supplementary Fig. 1 Protein expression analysis in genetically modified stem cell lines overexpressing selected miRNAs and unmodified controls. Proteins detected in hiPSC lines (left) and UC-MSCs (right).

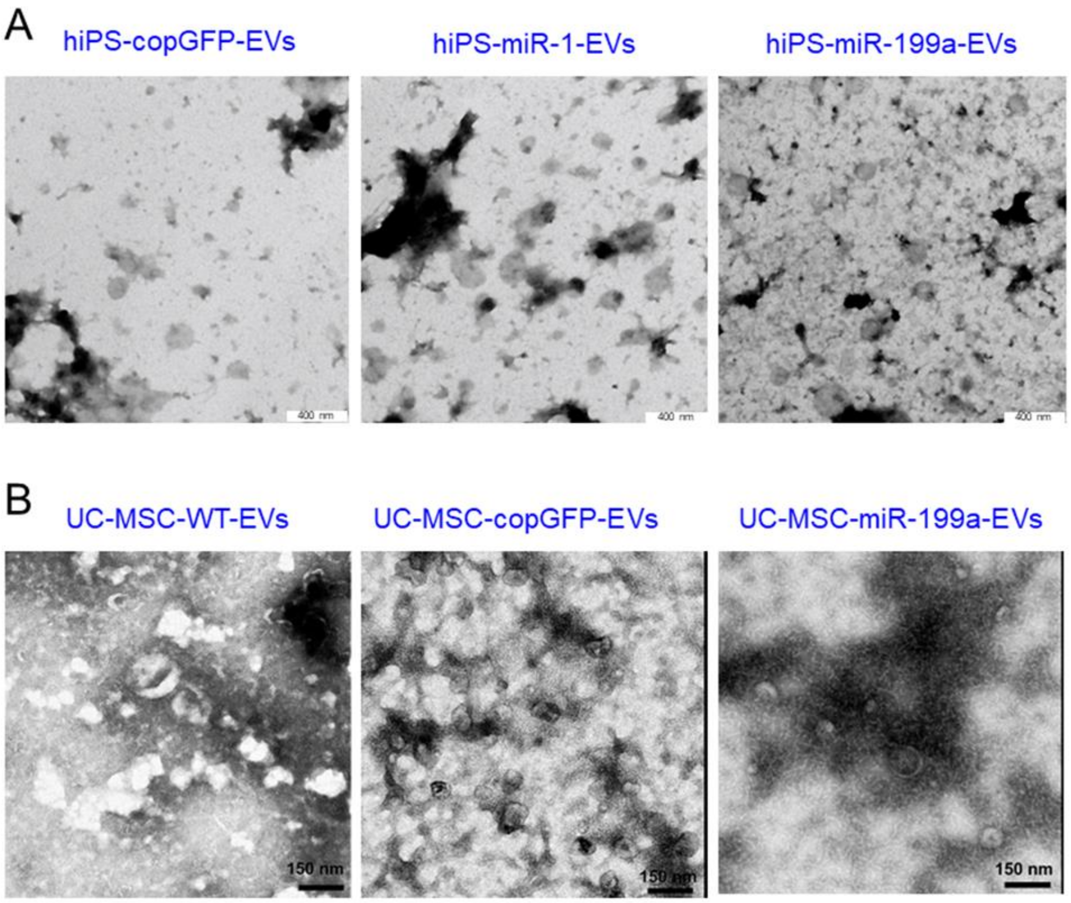

<u>Supplementary Fig. 2</u> TEM analysis of EVs isolated from hiPSC and UC-MSC lines overexpressing selected miRNAs and control cells.

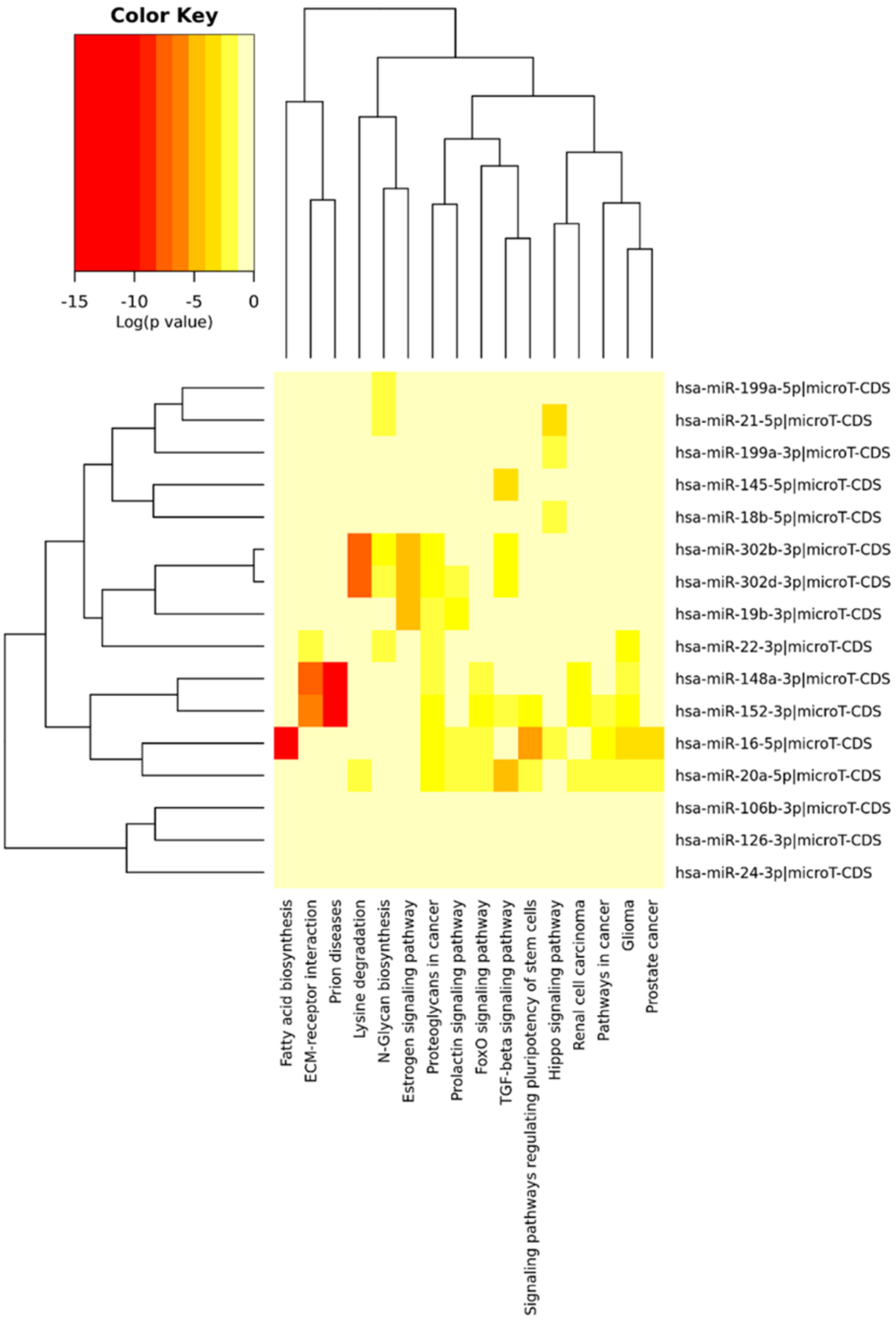

Supplementary Fig. 3 Pathways analysis of 16 miRNAs enriched in hiPS-miR-199a-EVs.

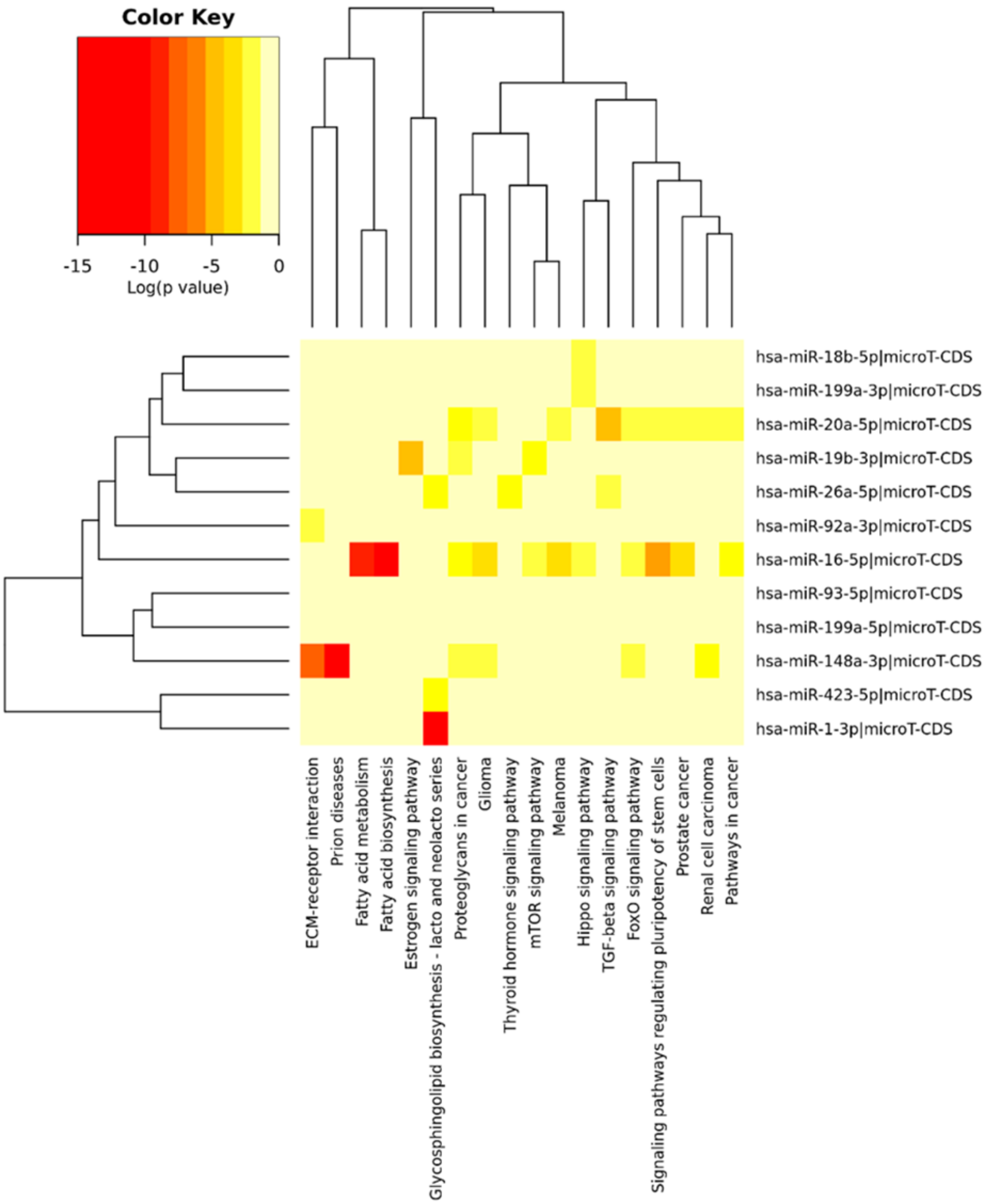

<u>Supplementary Fig. 4</u> Pathways analysis of 12 miRNAs enriched in <u>UC-MSC-miR-199a-EVs.</u>

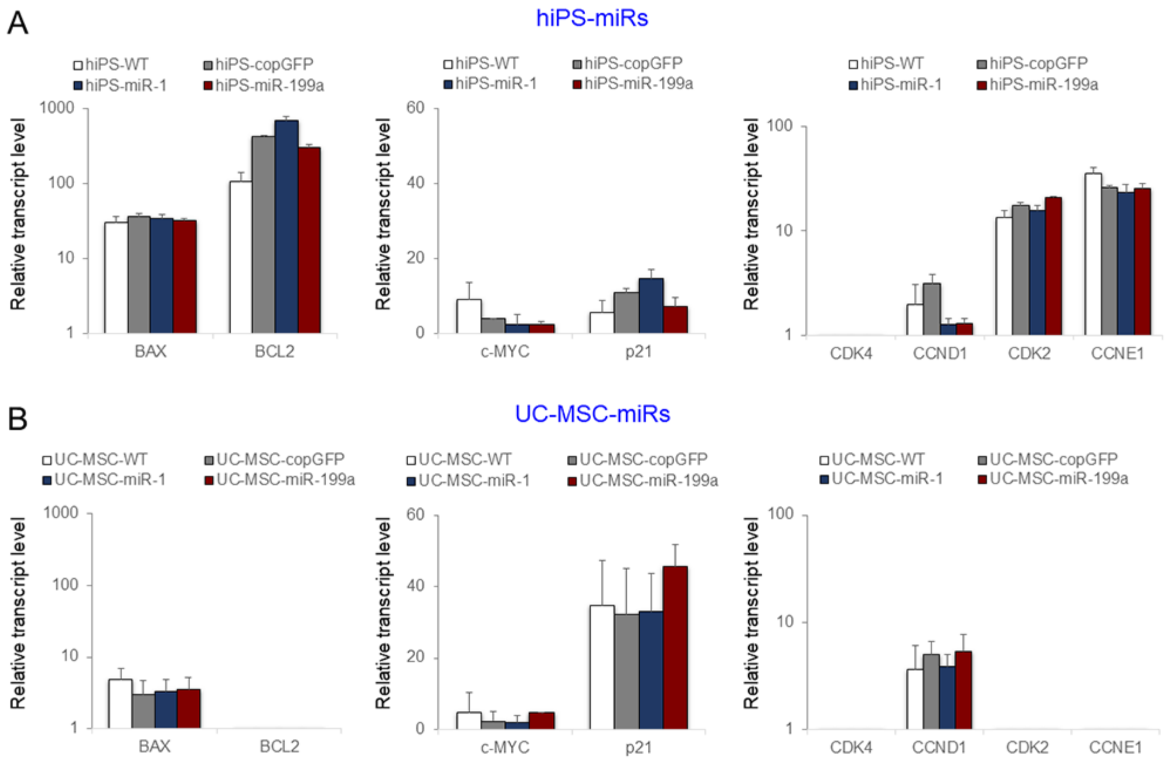

<u>Supplementary Fig. 5</u> Expression of genes regulating cell senescence in stem cel lines using RT-qPCR method. **(A)** Gene expression levels in hiPS-miRs cell lines. **(B)** Gene expression levels in UC-MSC-miRs cell lines.

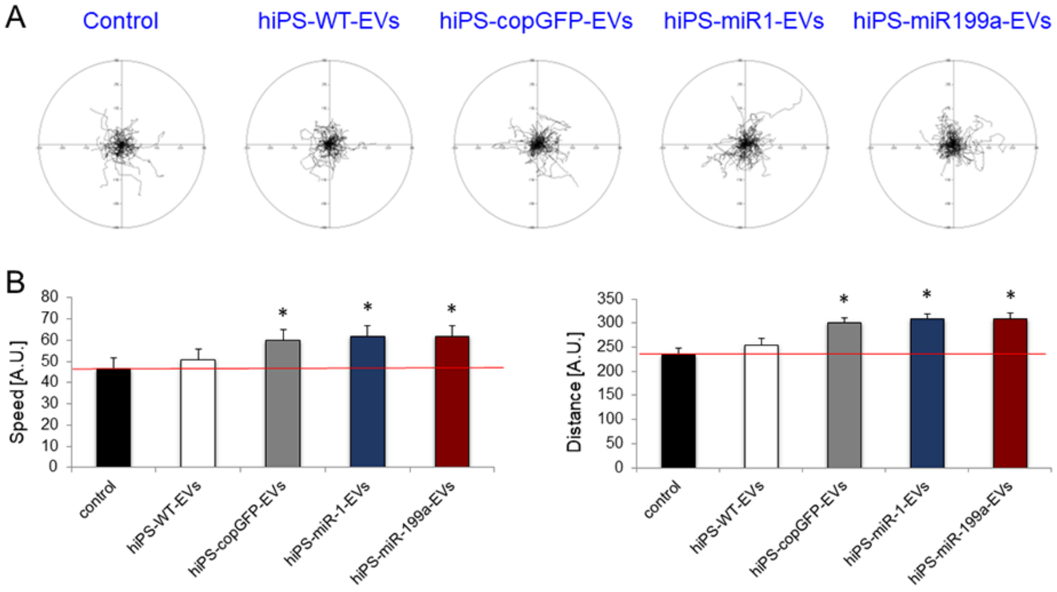

<u>Supplementary Fig. 6</u> Migration of CFs after EVs treatment. Migratory activity was recorded for 12h at 10-minute intervals. **(A)** Representative cell trajectories of fifty different CFs are shown (for each sample). **(B)** Quantitative data of cell speed (left panel) and distance (right panel) are presented. Control – CFs without EV treatment. Red bar indicates control level of both parameters. Student's t-test; *p<0.05

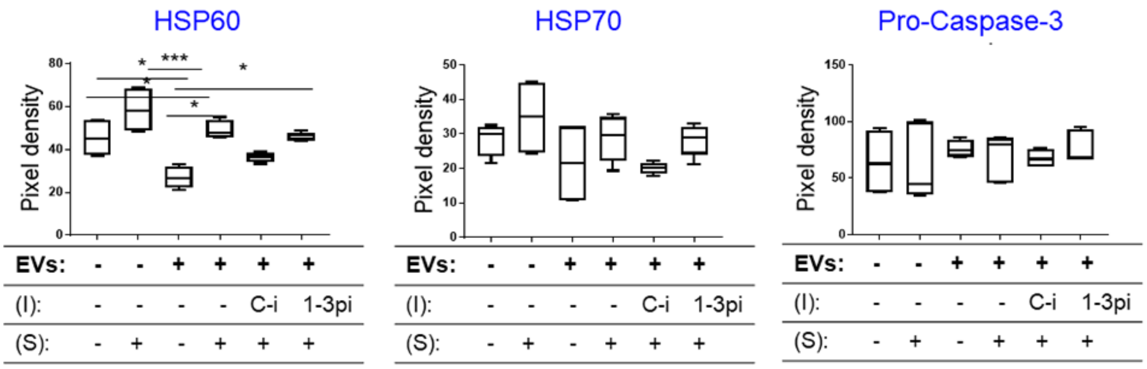

<u>Supplementary Fig. 7</u> Apoptosis pathway assessment in CFs after treatment with hiPS-miR-1-EVs. Protein array analysis of selected apoptosis- related proteins after incubation with EVs and cytotoxic agent (staurosporin; S), in the presence/absence of following miRNA inhibitors (I): specific miR-1-3p inhibitor (1-3p-i) or control inhibitor (C-i). ANOVA multiple comparisons and Tukey test; *p<0.05, **p<0.01, ***p<0.001.
